# Enzymatic diagnosis of Pompe disease: lessons from 28 years of experience

**DOI:** 10.1038/s41431-020-00752-2

**Published:** 2020-11-08

**Authors:** Monica Y. Niño, Mark Wijgerde, Douglas Oliveira Soares de Faria, Marianne Hoogeveen-Westerveld, Atze J. Bergsma, Mike Broeders, Nadine A. M. E. van der Beek, Hannerieke J. M. van den Hout, Ans T. van der Ploeg, Frans W. Verheijen, W. W. M. Pim Pijnappel

**Affiliations:** 1 Department of Pediatrics, Erasmus MC University Medical Center, Rotterdam, The Netherlands; 2 Department of Clinical Genetics, Erasmus MC University Medical Center, Rotterdam, The Netherlands; 3 Center for Lysosomal and Metabolic Diseases, Erasmus MC University Medical Center, Rotterdam, The Netherlands; 4 Department of Neurology, Erasmus MC University Medical Center, Rotterdam, The Netherlands

**Keywords:** Diagnosis, Disease genetics

## Abstract

Pompe disease is a lysosomal and neuromuscular disorder caused by deficiency of acid alpha-glucosidase (GAA), and causes classic infantile, childhood onset, or adulthood onset phenotypes. The biochemical diagnosis is based on GAA activity assays in dried blood spots, leukocytes, or fibroblasts. Diagnosis can be complicated by the existence of pseudodeficiencies, i.e., *GAA* variants that lower GAA activity but do not cause Pompe disease. A large-scale comparison between these assays for patient samples, including exceptions and borderline cases, along with clinical diagnoses has not been reported so far. Here we analyzed GAA activity in a total of 1709 diagnostic cases over the past 28 years using a total of 2591 analyses and we confirmed the clinical diagnosis in 174 patients. We compared the following assays: leukocytes using glycogen or 4MUG as substrate, fibroblasts using 4MUG as substrate, and dried blood spots using 4MUG as substrate. In 794 individuals, two or more assays were performed. We found that phenotypes could only be distinguished using fibroblasts with 4MUG as substrate. Pseudodeficiencies caused by the *GAA2* allele could be ruled out using 4MUG rather than glycogen as substrate in leukocytes or fibroblasts. The Asian pseudodeficiency could only be ruled out in fibroblasts using 4MUG as substrate. We conclude that fibroblasts using 4MUG as substrate provides the most reliable assay for biochemical diagnosis and can serve to validate results from leukocytes or dried blood spots.

## Introduction

Pompe disease is an autosomal recessive metabolic disorder caused by acid α-glucosidase (GAA) deficiency, which leads to intralysosomal accumulation of glycogen causing progressive damage especially to cardiac and skeletal muscles [1–3]. Pompe disease presents as a spectrum of phenotypes. The classic infantile form is associated with rapidly progressive general muscle weakness and hypertrophic cardiomyopathy, culminating in death within the first year of life if left untreated. Less severe and less progressive forms can manifest at any age from infancy to late adulthood and present with proximal muscle weakness and/or respiratory problems with minimal or no cardiac involvement [4–7]. These patients may eventually lose ambulation and/or become ventilator dependent. Patients with less severe forms and with the same *GAA* genotype can have broad phenotypic variation, with symptom onset ranging from early infantile to late adult [8]. This indicates the presence of environmental, epigenetic, or genetic modifying factors. Recently we identified the silent, cis-acting c.510C>T *GAA* variant as a genetic modifier of symptom onset and splicing in Pompe disease [9]. Symptoms in other tissues and organs also occur including in smooth muscle, visceral organs, and, in the classic infantile form, the central nervous system. Enzyme replacement therapy with recombinant human GAA is available and is often applied in combination with immunomodulation in classic infantile cases to reduce the chance of antibody formation [10–13]. Early diagnosis including phenotype prediction is required to optimize counseling and to ensure a timely start of treatment.

Current diagnostic guidelines recommend the establishment of GAA enzyme deficiency with additional confirmation of two disease-associated *GAA* variants [14, 15]. Disease-associated variants, recently linked to clinical phenotypes, are listed in the open access Pompe disease *GAA* variant database [www.pompevariantdatabase.nl] [16, 17]. Different enzymatic diagnostic assays are available in which the biological material and the choice of substrate are variables.

Biological materials can be leukocytes, dried blood spots (DBSs), fibroblasts derived from skin biopsies, and muscle tissue. Leukocytes and DBSs are easily obtained, which is important for timely screening, diagnosis, and treatment of classic infantile patients. Newborn screening (NBS) programs are based on DBS assays and positive cases require a second-tier assay for confirmation [18]. Muscle biopsies can be used for diagnosis however, these are not always available and involve a rather invasive and sometimes painful procedure. In addition, in the case of fat replacement of muscle, which can occur in Pompe disease, it can be difficult to obtain muscle cells from a muscle biopsy. Substrates include the natural substrate glycogen, and the artificial substrates 4-methylumbelliferyl-α-D-glucopyranoside (4MUG) [19] and (GAA-S), a substrate used in tandem mass spectrometry for NBS [20]. In certain assays such as using leukocytes or DBSs, the neutral hydrolase glucoamylase activity needs to be inhibited using acarbose as it interferes with the measurement of GAA activity [21–23].

Additional evidence for the diagnosis of Pompe disease can be obtained by measuring urine tetrasaccharide Glc4 (TGLC), which can be measured using HPLC with UV detection or using mass spectrometry. This biomarker has been found to be sensitive but it is not specific to Pompe disease, as it is also elevated in liver abnormalities or in response to food intake. In borderline cases, it might help to establish the diagnosis [24–26].

For the interpretation of diagnostic outcome, it is important to consider the following information on *GAA* DNA variants. (1) At least two (combinations of) *GAA* variants can lead to pseudodeficiency. In the Caucasian population, the *GAA2* (c.271G>A) pseudodeficiency variant lowers the activity of GAA for the natural substrate glycogen, but not for the artificial substrate 4MUG [27, 28]. In the Asian population, the common *GAA* pseudodeficiency variants c.[1726G>A; 2065G>A] can lower GAA activity to levels that come close to the disease threshold, and their presence can lead to a false positive diagnosis in certain assays [23, 29, 30]. (2) Standard DNA diagnostic analysis may fail to identify *GAA* variants, in these cases extended analysis is required as described [31–33].

Over the past 28 years, our laboratory has processed 1709 diagnostic cases and has diagnosed over 250 patients with Pompe disease using various assays separately and in parallel. In this article we are presenting and reviewing all our test results of blood-based and fibroblast-based assays from 1990 until 2018 allowing to compare the various methods for enzymatic diagnosis and their pitfalls.

## Materials and methods

### Nomenclature and Pompe variant database

The variant nomenclature is according to HGVS standards [34]. The reference sequences used for the annotation of *GAA* cDNA variants were NM_000152.5 and LRG_673t1.1. Position c.1 represents the first nucleotide of the translation start codon ATG located in exon 2. Exon numbering was according to Niño et al., 2019 [17]. NP_000143.2 was used for annotation of GAA protein variants. Variant information was retrieved from the Pompe disease *GAA* variant database at www.pompevariantdatabase.nl and the Leiden open variation database (http://lovd.nl/gaa).

### Diagnostic materials and assay procedures

GAA activity assays were performed when there was a suspicion of Pompe disease based on clinical symptoms (described below). Results were obtained over the period 1990–2018. Reference ranges reflect those of the Diagnostic Department of the Erasmus MC and were established for each assay based on at least 20 individuals per phenotype (classic infantile, childhood, adulthood, healthy controls) with a confirmed clinical diagnosis. Values in between these ranges are specified as ‘gray zone’, which is defined as GAA enzyme activities above the patient range but below the control range. For fibroblasts, the reference ranges were derived from data collected over a 40 years period. For the other assays, including GAA activity in leukocytes and bloodspots, and tetrasaccharide 6-α-D-glucopyranosyl-maltotriose (Glc4) concentration in urine by mass spectrometry, data collection of at least 8-years was used [24]. All reference ranges for patients, healthy controls, and the gray zones are indicated in the legends of the figures. The study was conducted according to the Declaration of Helsinki. The Medical Ethical Committee at Erasmus University MC approved the study protocol, and all patients, or their parents or legal guardians, provided written informed consent.

Activity assays were performed as previously described [22, 24, 35–37]. A final concentration of 3 µmol/L acarbose (in the reaction mixture) was used for the glycogen assay and 8 µmol/L acarbose for the 4MUG assay to inhibit glucoamylase activity present in mixed leukocytes and which interferes with GAA activity measurements. Performance of the assays was determined for each individual assay by including an internal standard sample. According to standard diagnostic procedures, a maximal deviation of less than 10% of the internal standard was required to use values as diagnostic values. For all assays, no false positive or false negative values of the internal standard were obtained. Typical standard deviations were: leukocytes/4MU: 5.1% (*n* = 29); leukocytes/glycogen: 6.2% (*n* = 72); fibroblasts/4MUG: 7.1% (*n* = 78); DBS (in our Diagnostic Center not used as diagnostic assay): 11.3% (*n* = 7). The GAA activity in DBS was determined in the presence of 8 µmol/L acarbose for glucoamylase inhibition and after precipitation of hemoglobin as previously described [35, 36].

### Clinical diagnosis

We classified patients with classic-infantile Pompe disease when they presented symptoms at or under 12 months of age. Symptoms in these patients consist of muscle weakness combined with a hypertrophic cardiomyopathy. The phenotype of childhood and adulthood onset Pompe disease is dominated by a progressive limb-girdle myopathy that leads to severe functional limitations, while respiratory muscle dysfunction limits patients’ average life span. Childhood onset is here defined as symptom onset before the age of 18 years, adult onset at 18 years or older. The diagnosis was established based on combined evidence consisting of these clinical symptoms, GAA enzyme deficiency, and DNA analysis.

## Results

**Fig. 1:**
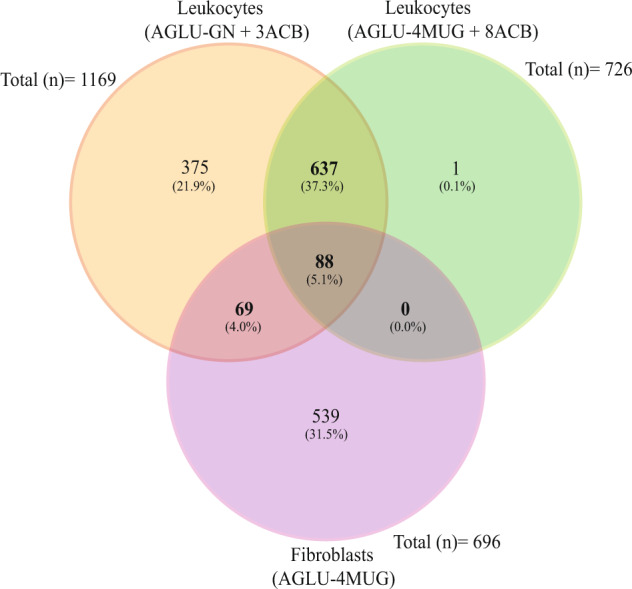
Venn diagram showing how many diagnostic cases were processed with each of the three different enzymatic methods. The figure shows the similarities and differences of individuals whereby the GAA activity was measured in leukocytes with glycogen as well as with 4MUG as substrate, and in fibroblast with 4MUG. Of note 11 assay results (five from leukocytes with glycogen four from leukocytes with 4MUG, and two from fibroblast with 4MUG) were not included in this comparison since they are biological replicates. DBS assays (20 assays from 18 individuals) are not shown.

Over the past 28 years, we analyzed a total of 1709 individuals using a total of 2591 assays (Fig. 1 and Table S1). In the majority of individuals, leukocytes were used with both glycogen and 4MUG as substrate (*n* = 637). In 539 individuals, fibroblasts using 4MUG as substrate were used as single assay, while in 375 individuals, leukocytes with glycogen were used as single assay. Only one individual was analyzed with leukocytes using 4MUG as substrate as single assay. Eighty-eight individuals were analyzed with all three assays, while 69 individuals were analyzed with both leukocytes/glycogen and fibroblasts/4MUG. As part of this study we traced back the clinical data of all patients with a confirmed enzymatic diagnosis. The following categories for the diagnoses were defined: ‘Classic Infantile’, ‘Childhood onset’ (symptom onset before the age of 18 years), ‘Adult onset’ (symptom onset at 18 years and older), ‘no Pompe disease’, and ‘Unknown’. For enzymatic assays, the following additional categories were defined: ‘Unknown/Deficient’ (i.e., the value of the assay was deficient but it was unknown whether the individual had Pompe disease), ‘No Pompe/Deficient’ (i.e., the value of the assay was deficient but Pompe disease was excluded based on other assays), ‘Asymptomatic/Deficient’ (i.e., the value of the assay was deficient but the individual was asymptomatic), and ‘Gray Zone’ (the values above patient ranges but below normal ranges).

### GAA activity assay in leukocytes using glycogen as substrate

**Fig. 2:**
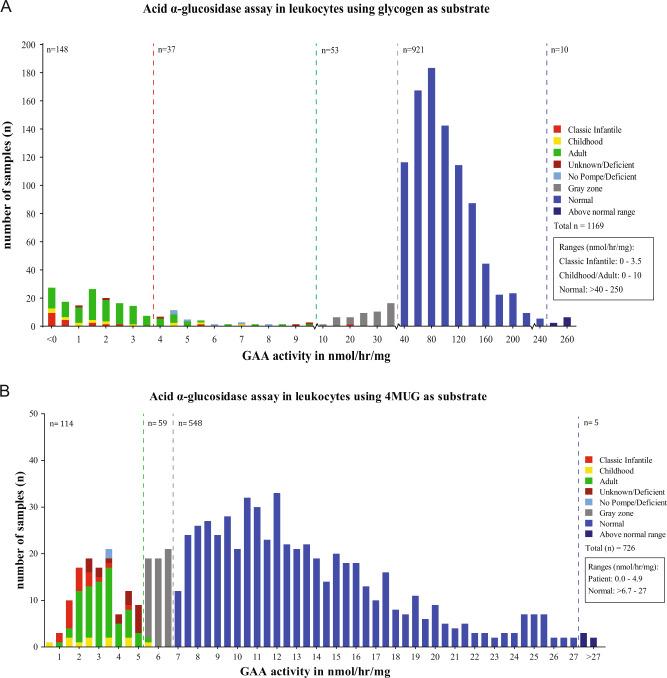
Distribution of GAA activity in leukocytes using. **A** Glycogen as substrate in the presence of 3 µmol/L acarbose (AGLU-GN+ 3ACB assay). A total of 1169 individuals suspected of Pompe disease were included. **B** 4MUG as substrate in the presence of 8 µmol/L acarbose (AGLU-4MUG+ 8ACB assay). A total of 726 individuals suspected of Pompe disease were included in this series.

Figure 2A shows GAA activities using glycogen as substrate that were measured in 1169 individuals. Within the range of 0–3.5 nmol/mg/h for classic infantile patients, there were 20 classic infantile patients, 15 with childhood onset, and 111 with adult onset Pompe disease. Two individuals were ‘unknown deficient’. Within the range of 3.5–10 nmol/mg/h, which is the higher activity range for childhood/adult onset patients (range 0–10 nmol/h/mg), there were 1 classic infantile patient, 6 with childhood onset, 20 with adult onset, 7 ‘no Pompe/deficient’, and 3 ‘unknown/deficient’. This indicated that the range of 0–3.5 nmol/mg/h contained 95% of the classic infantile, 71% of the childhood onset, and 84% of the adulthood onset patients. The 3.5–10 nmol/mg/h range contained 5% of the classic infantile, 29% of the childhood onset, and 16% of the adulthood onset patients. In both ranges, putative false positives were found to a total of 12 individuals. A subset of these false positives was followed up using leukocytes/4MUG and fibroblasts/4MUG assays, and these showed mainly gray zone (seven assays) and normal (nine assays) values, while one assay in leukocytes/4MUG showed an unknown/deficient value (see Table S1 for raw data). Within the range of 10–40 nmol/mg/h, 52 individuals were classified with gray zone (above the patient range but below the control range) values, while 1 classic infantile patient was present. This patient showed values in the patient range in leukocytes/4MUG (3.46 nmol/mg/h) and in the classic infantile range in fibroblasts/4MUG (0.2 nmol/mg/h). In the normal ranges above 40 nmol/mg/h, a total of 921 healthy individuals were present. In conclusion, the leukocytes/glycogen assay has limited ability to discriminate between patients with classic infantile and late onset phenotypes, has a considerable risk of false positive, and a low risk of false negative outcomes.

### GAA activity assay in leukocytes using 4MUG as substrate

In Fig. 2B, the activities in leukocyte assays using 4MUG as substrate, measured in 726 individuals, are shown. In the patient range of 0.0–4.9 nmol/h/mg, there were 19 classic infantile patients, 10 with childhood and 66 with adulthood onset, 2 were no Pompe/deficient, and 17 were unknown/deficient. The two individuals that were no Pompe/deficient and the 17 individuals that were unknown/deficient showed either gray zone and/or normal values when tested in leukocytes/4MUG and/or fibroblasts/4MUG. This showed that the leukocyte/4MUG assay did not distinguish classic infantile from late onset phenotypes. The range 4.9–6.7 showed 1 childhood onset patient, 1 adult onset patient, and 57 gray zone individuals. The childhood onset patient had borderline activity just above the threshold (5.14 nmol/mg/h) and tested in the patient range (0.365 nmol/mg/h) in the leukocyte/glycogen assay. The healthy range > 6.7 nmol/mg/h included 548 healthy individuals and 5 individuals with somewhat increased enzyme activities. This indicated that the leukocyte/4MUG assay could faithfully discriminate between healthy and diseased individuals but could also result in false positive outcomes.

### GAA activity assay in fibroblasts with 4MUG as substrate

**Fig. 3:**
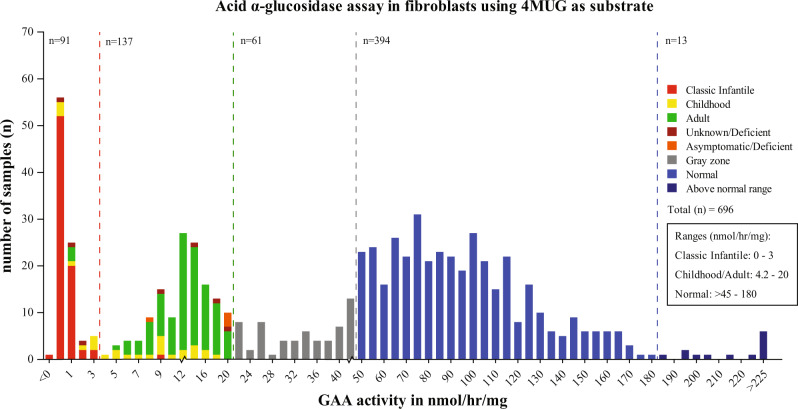
Distribution of GAA activity in fibroblast using the artificial substrate 4-methylumbelliferyl-α-D-glucoside (4MUG) (AGLU-4MUG assay). A total of 695 individuals suspected of Pompe disease were included in this series.

In Fig. 3, the activities in fibroblasts using 4MUG as substrate are shown. In the range 0–3 nmol/h/mg for classic infantile patients, there were 77 classic infantile patients, 8 patients with childhood and 3 with adulthood onset. One patient with childhood onset had an activity in between 3 and 4.2. In the range 4.2–20 nmol/mg/h for childhood/adult onset patients, there were 1 classic infantile patient, 18 with childhood onset and 108 with adulthood onset. There were six asymptomatic/deficient individuals. These individuals may develop symptoms later in life, as symptom onset in Pompe disease can be highly variable. Two of these individuals were also tested using leukocytes/glycogen and 4MUG, and in both individuals, these two assays yielded gray zone values. This indicated that the fibroblasts/4MUG assay could distinguish classic infantile from childhood/adulthood onset patients to a large extent, and between childhood and adulthood patients to a lesser extent. There were 61 individuals with enzyme activities in the gray zone range of >20–45  nmol/h/mg. All individuals with activities in the healthy range >45 nmol/h/mg were healthy. This showed that the fibroblast/4MUG assay can distinguish both healthy from diseased individuals and classic infantile from childhood/adulthood onset phenotypes.

### GAA activity assay in DBS

Table 1 shows the results of DBS assays in 18 individuals with Pompe disease. In 15 cases (12 classic infantile, 2 childhood, 1 with an unknown phenotype), GAA activity was in the patient range of 11–56 pmol/17 h/punch, while three individuals (patients 30–32, who had classic infantile Pompe disease) were in the gray zone of 56–94 pmol/17 h/punch. However, all individuals including individuals 30–32 tested in the patient range in leukocytes with glycogen as substrate. One sample, from patient 32 was measured three times, of which one was in the patient range and 2 in the gray zone. Fibroblasts from this patient showed activity in the patient range and DNA analysis of this patient and patients 30 and 31 confirmed the presence of two severe *GAA* disease-associated variants (Table 2 and Fig S1). These results indicated that in this cohort the DBS assay resulted in relatively frequent false negative outcomes (16.7% in this analysis) although the number of DBS samples analyzed here (*n* = 18) was low.

**Table 1:** GAA activity in Pompe patients measured in DBS and leukocytes.

Patient	Dried blood spot (DBS)		LEUKOCYTES		Clinical diagnosis
	AGLU-4MUG + 8ACB (pmol/17 h/punch)	BGAL (pmol/17 h/punch)	AGLU-GN+3ACB (nmol/h/mg)	BGAL (nmol/h/mg)	
15	*30.8*	4730	*1.6*	152	Classic infantile Pompe disease
16	*48.8*	2480	*1.0*	131	Childhood onset Pompe disease
17	*13.8*	2860	*–0.1*	162	Classic infantile Pompe disease
18	*25.2*	2520	*0.8*	129	Classic infantile Pompe disease
19	*17.4*	1660	*5.0*	147	Classic infantile Pompe disease
20	*11.4*	1380	*1.2*	192	Classic infantile Pompe disease
21	*10.6*	1780	*–1.1*	174	Classic infantile Pompe disease
22	*27.8*	3100	*–0.2*	192	Classic infantile Pompe disease
23	*14.6*	2040	*–1.6*	167	Classic infantile Pompe disease
24	*12.9*	2950	*0.3*	207	Classic infantile Pompe disease
25	*25.4*	3920	*0.1*	172	Classic infantile Pompe disease
26	*28.6*	3710	*2.8*	104	Childhood onset Pompe disease
27	*25*	4990	*0.9*	222	Unknown
28	*8.51*	3060	*1.2*	161	Classic infantile Pompe disease
29	*41.5*	4700	*–0.4*	181	Classic infantile Pompe disease
30	59.4	3620	*–2.0*	225	Classic infantile Pompe disease
31	58.6	6050	*–1.1*	169	Classic infantile Pompe disease
32*	85.3	4710	*–0.7*	139	Classic infantile Pompe disease
	68	5230			Classic infantile Pompe disease
	*48.6*	3250			Classic infantile Pompe disease
Normal range	94–448	476–4680	>40–250	50–326	
Patient range	11–56	88	Classic infantile: 0–3.5	0.6–6.3	
			Childhood/Adult: 0–10		

Cases 30–32 were borderline inconclusive using the DBS, but were positively confirmed with leukocytes as sample source. *Patient 32 showed variable measurements of GAA activity and BGAL in DBS (technical replicates were performed). Patient 16 and 26 were diagnosed with childhood Pompe disease, and individual 27 was classified as Unknown/Deficient due lack of clinical information.

Italic: values within the patient range.

**Table 2:** Follow up of three cases with inconclusive DBS data.

Patient	Genotype DNA (protein)	Predicted severity (Pompe disease *GAA* variant database)	ACMG variant classification	Fibroblast (GAA activity)	Clinical diagnosis
				AGLU-4MUG (nmol/h/mg)	
30	c.1460T>C p.(Phe487Ser);	Potentially less severe	Likely pathogenic	0.432	Classic infantile
	c.1460T>C p.(Phe487Ser)	Potentially less severe	Likely pathogenic		Pompe disease
31	c.379_380del p.(Cys127Leufs*18);	Very severe	Pathogenic	0.35	Classic infantile
	c.525del p.(Glu176Argfs*45)	Very severe	Pathogenic		Pompe disease
32	c.2481+102_2646+31del p.(Gly828_Asn882del);	Very severe	Pathogenic	0.415	Classic infantile
	c.525del p.(Glu176Argfs*45)	Very severe	Pathogenic		Pompe disease
Ranges (GAA activity)		Normal range		>45–180	
		Classic infantile		0–3	
		Childhood/Adult		4.2–20	

DNA analysis and GAA activity in cultured fibroblasts left no doubt that all three patients had classic infantile Pompe disease.

### GAA2 pseudodeficiency

Individuals with pseudodeficiencies can give false positive outcomes in GAA enzyme assays. To illustrate this, we compared individuals with confirmed presence of the *GAA2* (c.271G>A) and Asian c.[1726G>A; 2065G>A] pseudodeficiencies using different GAA activity assays.

In four individuals with the *GAA2* variant at heterozygous state without other known GAA disease-associated variants, activities were in the gray zone (two cases) or in the normal range (two cases) in leukocytes/glycogen (Table 3). In leukocytes/4MUG, one case had activity in the gray zone, and three in the normal range. In fibroblasts/4MUG, three patients were tested, two of whom were in the normal range and one (individual 4) in the patient range. This indicated that the *GAA2* allele at heterozygous state can already lower GAA activity in leukocyte-based assays to values in the gray zone. Individual 4 had a medical record of hypertrophic cardiomyopathy. This individual underwent metabolic screening as no cause for his hypertrophic cardiomyopathy could initially be found. Genetic testing using WES led to the identification of a disease-associated variant in exon 19 (c.1831G>A, p.Gly611Ser) of the *MYBPC1* gene (Myosin Binding Protein C, slow type), which plays an important role in muscle contraction and cardiac conduction. No muscle biopsy or MRI was performed, CK was normal (91 U/l). He had an incomplete traumatic cervical spinal cord lesion; he can walk but suffers from spasms due to this condition. General and neurological examination showed no other abnormalities, and there were no signs or symptoms of Pompe disease.

**Table 3:** GAA enzyme activity in leukocytes and fibroblast from individuals carrying the *GAA2* pseudodeficiency allele (c.271G>A p.(Asp91Asn).

Individual/patient (gender)	Genotype DNA (protein)	Enzymatic assay			Clinical diagnosis
		Leukocytes		Fibroblast	
		AGLU-GN + 3ACB	AGLU-4MUG+ 8ACB	AGLU-4MUG	
		(nmol/h/mg)	(nmol/h/mg)	(nmol/h/mg)	
1 (M)	c.271G>A p.(Asp91Asn)	36.2	7.9	61.7	No Pompe disease
2 (M)	c.271G>A p.(Asp91Asn)	46.6	8.5	50.4	No Pompe disease
3 (M)	c.271G>A p.(Asp91Asn)	77.6	12.2	N.D.	No Pompe disease
4 (M)^a^	c.271G>A p.(Asp91Asn)	24.0	5.7	*19.5*	No Pompe disease
5 (M)	c.271G>A p.(Asp91Asn); c.271G>A p.(Asp91Asn)	*7.6*	10.9	49.8	No Pompe disease
6 (M)^b^	c.271G>A p.(Asp91Asn); c.271G>A p.(Asp91Asn)	*4.4/7.7*	11.1	86.9	No Pompe disease
7 (F)	c.271G>A p.(Asp91Asn); c.-32-13T>G p.(=), p.(0)	*2.2*	6.1	25.9	No Pompe disease
8 (M)	c.271G>A p.(Asp91Asn); c.-32-13T>G p.(=), p.(0)	*4.1*	N.D.	51.0	No Pompe disease
9 (F)	c.271G>A p.(Asp91Asn); c.-32-13T>G p.(=), p.(0)	*4.7*	6.5	53.5	No Pompe disease
10 (F)	c.271G>A p.(Asp91Asn); c.-32-13T>G p.(=), p.(0)	*6.6*	N.D.	53.1	No Pompe disease
11 (F)^c^	c.271G>A p.(Asp91Asn); c.-32-13T>G p.(=), p.(0); c.1076-22T>G p.?	*5.7*	N.D.	33.3	No Pompe disease
12 (F)	c.271G>A p.(Asp91Asn); c.-32-13T>G p.(=), p.(0); c.1447G>A p.(Gly483Arg)	*1.5*	*1.9*	*7.6*	Adult onset Pompe disease
Ranges	Normal range	>40–250	>6.7–27	>45–180	
	Patient range	Classic infantile: 0–3.5	1.1–4.9	Classic infantile: 0–3	
		Childhood/Adult: 0–10		Childhood/Adult: 4.2–20	

Italic: values within the patient range.

^a^Unaffected by Pompe disease, but a disease-associated variant was found in the MYBPC1 gene, which is associated with cardiac hypertrophy.

^b^The biological replicate was 7.7 nmol/h/mg in leukocytes using glycogen as substrate.

^c^Unaffected by Pompe disease.

In individuals that were homozygous for *GAA2* (number 5 and 6), GAA activities were in the patient range in leukocytes/glycogen, but in the normal range in leukocytes/4MUG and in fibroblasts/4MUG (Table 3). In individuals that were compound heterozygous for *GAA2* in combination with a second disease-associated *GAA* variant (numbers 7–11), GAA activities were in the patient range in leukocytes/glycogen in all cases (individual 11 contained an additional *GAA* variant classified as childhood when combined with a null allele according to www.pompevariantdatabase.nl) (Table 3). In leukocytes/4MUG and fibroblasts/4MUG, values were either in the gray zone or in the normal range. This highlights that the *GAA2* pseudodeficiency at homozygous or compound heterozygous state can give false positive outcomes when using the leukocyte/glycogen assay, while correct outcomes can be obtained by using either the leukocytes/4MUG or fibroblast/4MUG assays. Patient 12 contained 2 *GAA* disease-associated variants in addition to the *GAA2* allele at heterozygous state, and showed GAA values in the patient range in all three assays consistent with the diagnosis of Pompe disease (adulthood onset).

### Asian c.[1726G>A; 2065G>A] pseudodeficiency

Individual 13 was compound heterozygous and individual 14 homozygous for the Asian c.[1726G>A; 2065G>A] pseudodeficiency (Table 4). In both cases, assays using leukocytes, either with glycogen or with 4MUG as substrate, resulted in activities in the patient range, while activities in fibroblasts/4MUG were in the gray zone, just above the patient range. Additional diagnostic analyses showed that individual 13 had normal CK, ASAT, and ALAT levels, but slightly elevated TGLC levels. Individual 14 did have elevated CK, ASAT, and ALT levels but normal TGLC levels. Both individuals were diagnosed not to have Pompe disease based on the diagnostic information and the lack of clinical signs associated with Pompe disease. This indicated that the presence of the Asian pseudodeficiency can seriously affect the diagnostic outcome of enzymatic assays that are based on leukocytes, independent of the substrate used.

**Table 4:** Lack of diagnosis in cases with the Asian pseudodeficiency (*c.1726G*>*A and c.2065G*>*A)*.

Individual (gender, age in years)	Genotype DNA (protein)	Enzymatic assay			Ancillary studies				Clinical diagnosis
		Leukocytes		Fibroblast	Plasma			Urine	
		AGLU-GN+ 3ACB	AGLU-4MUG+8ACB	AGLU-4MUG	CK	ASAT	ALAT	TGLC	
		(nmol/h/mg)	(nmol/h/mg)	(nmol/h/mg)	(U/l)	(U/l)	(U/l)	(mmol/mol creatinine)	
13 (F, 74 years)	c.1726G>A p.(Gly576Ser); c.2065G>A p.(Glu689Lys); c.-32-13T>G p.(=), p.(0)	*9.6*	*3*	22	58–104	27–34	19–32	*2.3*^a^	No Pompe disease
14 (M, 53 years)	c.1726G>A p.(Gly576Ser); c.2065G>A p.(Glu689Lys); c.1726G>A p.(Gly576Ser); c.2065G>A p.(Glu689Lys)	*18.7*	*3.9*	20.75	992	50	57	0.8	No Pompe disease
Ranges	Normal range	>40–250	>6.7–27	>45–180	W > 17 years: <170	W > 17 years: <31	W > 17 years: <31	>20 years: 0–2.2	
					M > 17 years: <200	M > 17 years: <37	M > 17 years: <41		
	Patient range	Classic infantile: 0–3.5	1.1–4.9	Classic infantile: 0–3				>20 years: 2.3–130	
		Childhood/Adult: 0–10		Childhood/Adult: 4.2–20					

GAA activities were measured both in leukocytes using the natural and artificial substrates as well as in cultured fibroblasts.

Italic: values within the patient range.

^a^2th measurement of TGLC was 5.1 and a 3rd measurement was 11.6 mmol/mol creatinine.

## Discussion

We compared the outcome of different assay procedures for measuring the GAA activity in various sample types using different substrates measured at the Erasmus MC in the last 28 years. The pro’s and con’s of the different diagnostic procedures are discussed below.

### Blood-based assays

#### Leukocytes

**Fig. 4:**
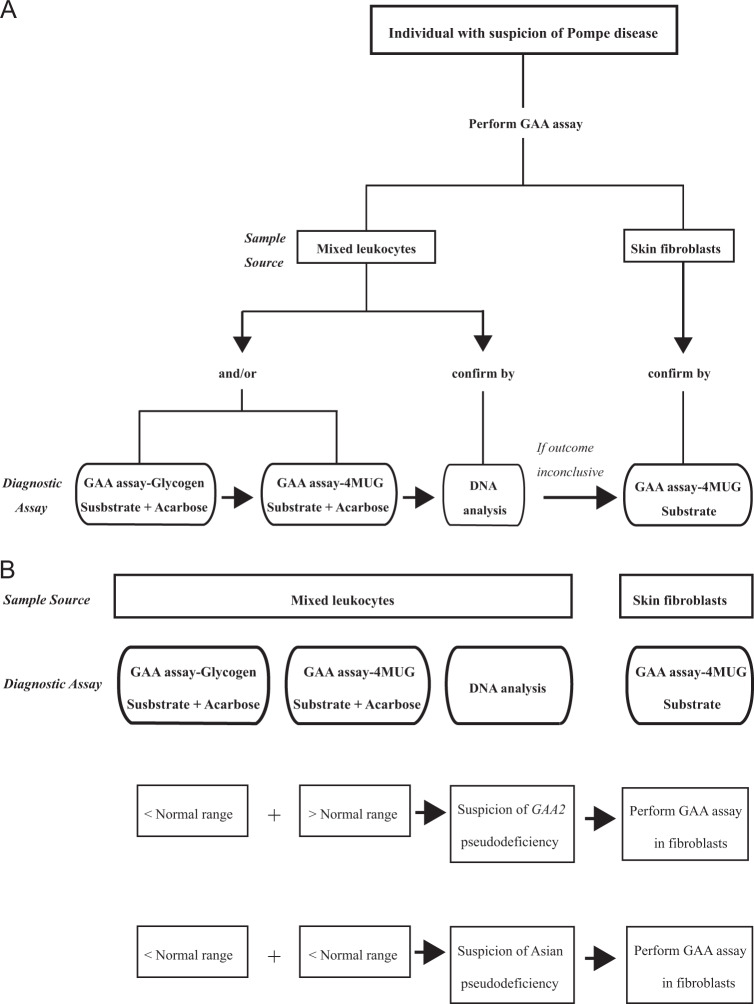
Flow chart for diagnosis of Pompe disease. **A** General flow chart. **B** Flow chart for cases with pseudodeficiency.

We used leukocyte pellets for diagnostic purposes and applied both glycogen as well as 4MUG as substrate [36]. The results presented in Fig. 2A, B illustrate that a diagnosis can be established in most cases and with both substrates if proper cutoff values are chosen. As previously suggested by van Diggelen et al. [36], our long-term data also demonstrate that the dynamic range, i.e., the difference in values between patients and healthy individuals, is broader with glycogen than with 4MUG. None of the two assays fully discriminate between classic and milder (childhood, adult) phenotypes, but by using both assays in parallel, pseudodeficiencies caused by the Caucasian *GAA2* allele can be excluded (Fig. 4B) [27, 28]. Another pseudodeficiency that complicates the assay is the Asian c.[1726G>A; 2065G>A] pseudodeficiency [23, 29, 30]. This problem applies to all enzymatic procedures used.

#### Bloodspots

In our small patient cohort, 3 (16.7%) of 18 patients (assays 30–32 in Fig. S1) came out as false negative in the DBS assay but were correctly diagnosed with other diagnostic methods. Analysis of the activity of a second lysosomal enzyme (for instance BGAL) as reference enzyme may help to judge the outcome of the assay. For example, a normal GAA activity should not be trusted if the reference enzyme shows activity outside of the normal ranges. In such an event additional assays are required. In our diagnostic center we do not use the DBS assay routinely as we have fast and standard procedures for leukocyte-based assays. DBS-based assays prove to be very valuable tools for NBS. In both individual cases and in NBS, a second assay is required to confirm the diagnosis.

Despite the ten times higher activity of GAA for glycogen compared to 4MUG, the small sample size of the bloodspot assay precludes the use of glycogen for DBS testing due to the lower sensitivity of the colorimetric assay used with glycogen compared to the fluorimetric detection using 4MUG. The tandem mass spectrometry methodology, employing GAA-S has proven to be a valuable alternative [20]. A recent refinement of that method was reported to separate 96% of the Taiwanese newborns with GAA pseudodeficiency and all Pompe disease carriers from patients with Pompe disease [38]. A recent study from Japan supports this claim [39].

#### Skin fibroblast assays

Figures 2A, B and 3 clearly demonstrate that the combination of fibroblasts as diagnostic material and the fluorimetric assay using 4MUG as artificial substrate provide the most robust and reliable enzymatic assay for diagnosing Pompe disease. In the majority of cases it distinguishes classic infantile Pompe disease from childhood/adulthood onset phenotypes on the basis of residual activity (although exceptions exist), while none of the other methods do. Assays with intermediate GAA activity were likely derived from Pompe disease carriers. DNA analysis usually provides a clear answer in these cases, but some remained unsolved. For instance, in individual 13, that carried the Asian pseudodeficiency c.[1726G>A; 2065G>A] in combination with the IVS1 variant, biochemical results suggested Pompe disease while clinical signs did not, but it leaves doubt whether adult/late onset Pompe disease can be fully ruled out in this patient.

## Conclusions and recommendations

We conclude from our data that cultured skin fibroblasts provide the only sample source that can distinguish classic infantile from childhood/adulthood phenotypes, although exceptions can occur, as noted previously [40]. The laborious procedure of biopsy and culturing makes this assay not suitable for a fast diagnosis. Leukocytes isolated from peripheral blood offer a fast diagnostic sample source, provided that the reaction mixture contains acarbose to inhibit glucoamylase. The use of glycogen as natural substrate enhances the resolution between affected and unaffected, but *GAA2* pseudodeficiency occurs in the Caucasian population and has to be excluded by also using 4MUG as substrate in case of exceptionally low GAA activity (Fig. 4B). The implementation of DBSs has enabled (newborn) screening, and several analytical methods have proven their value as first-tier test. There is general agreement that additional tests remain necessary to finally establish the diagnosis. For reasons mentioned in the text, we advise to conduct genetic testing in addition to any type of enzymatic testing, as recommended recently by the EPOC consortium and others [18, 41].

The recommended enzymatic and molecular diagnostic flow at our Center is as follows: (1) start with leukocytes using 4MUG and glycogen as substrate; (2) confirm with DNA analysis, in which two disease-associated variants should be identified, while a pseudodeficiency does not qualify as disease-associated variant; (3) if there is doubt, for example when values are close to the gray zone or when a DNA variant is unidentified or novel with unknown significance unknown, use fibroblasts as confirmation (Fig. 4A, B).

## Supplementary information

Supplemental text

S1

Table S1
