# Supplementary material for: Enzymatic diagnosis of Pompe disease: lessons from 28 years of experience
Source: Eur J Hum Genet. 2020 Nov 8;29(3):434–46. doi: 10.1038/s41431-020-00752-2 (PMC7940434; doi:10.1038/s41431-020-00752-2)
Supplement: Supplementary file 1 — Supplemental text [file 41431_2020_752_MOESM1_ESM.pdf]

Supplementary Data

Supplementary text

Pseudodeficiencies and borderline cases

c.271G>A (GAA2): common Caucasian background

Table 3 lists individuals that contained the *GAA2* pseudodeficiency allele. In individuals 1-4, only the *GAA2* variant was detected in heterozygous state but no other disease-associated *GAA* variant were detected. GAA enzyme activities were generally low and in some cases in the gray zone, but above the patient range in all three assays (leukocytes using 4MUG or glycogen as substrate and fibroblasts using 4MUG as substrate), except for individual 4. This person showed an activity that was within the patient range in fibroblasts using 4MUG a substrate (19.5 nmol/mg/hr, patient range 4.2-20), while the activity was slightly above the disease threshold in leukocytes using 4MUG as substrate. This individual was diagnosed not to have Pompe disease. The genetic testing led to the identification of a disease-associated variant in exon 19 (c.1831G>A, p.Gly611Ser) of the MYBPC1 gene (Myosin Binding Protein C, Slow Type). The GAA enzymatic diagnostic outcome remained enigmatic.

Individual 5 and 6 were homozygous for *GAA2*. In both cases, the activity for glycogen was within the patients' range, but for 4MUG in the normal range. Also based on the normal activity in cultured fibroblasts, both cases were diagnosed not to have Pompe disease.

Individual 7-10 were compound heterozygous for *GAA2* and c.-32-13T>G (IVS1). Also in these cases, the activities using glycogen as substrate were in the patient range, but activities using 4MUG as substrate were in the normal range or gray zone.

Individuals 11 and 12 contained the *GAA2* allele in heterozygous state in addition to two disease-associated variants. Case 11 concerned an unaffected of six children, two females of whom have the adult Pompe disease phenotype. DNA analysis of the mother revealed the presence of two disease-associated variants on one *GAA* allele c.[-32-13T>G;

1076-22T>G] and the *GAA2* variant on the other. The activity in leukocytes was in the patient range when measured with glycogen as substrate, but in the gray zone (33.3 nmol MU/hr/mg) when measured in fibroblasts with 4MUG as substrate . She was diagnosed not to have Pompe disease. Case 12 demonstrated GAA deficiency in all three assays and was diagnosed with adult onset of Pompe disease. She contained [c.271G>A; c.-32-13T>G on one allele, and the disease-associated variant c.1447G>A p.(Gly483Arg) on the other. This was supported by her clinical history, mentioning unbalanced walking at the age of 26 years, progressing to difficulty with running, respiratory involvement at the age of 43, and weak back and walking difficulties one decade later. She developed walking disability and started ERT at the age of 64 years.

In conclusion, the pseudodeficiency *GAA2* allele can lower GAA enzyme activity measurements when using glycogen as substrate, but not when using 4MUG as substrate. This can result in values within the patient range when using leukocytes with glycogen as substrate in patients that carry a disease-associated variant on one allele and *GAA2* on the second allele.

c.1726G>A and c.2065G>A: common Asian background

Individual 13 was referred at the age of 73 and was compound heterozygous for c.[1726G>A, c.2065G>A] and c.-32-13T>G (IVS1) (Table 4). Activities in leukocytes were in the patient range, both when using glycogen and 4MUG as substrate. The activity in fibroblasts was just above the patient range in the gray zone. This individual also had elevated urinary glucose tetrasaccharide levels, but no pathology in a muscle biopsy. Individual 14 was referred at the age of 60 and was homozygous for c.[1726G>A, c.2065G>A]. GAA enzyme activity was in the patient range in leukocytes with 4MUG as substrate but not with glycogen as substrate; in fibroblasts the activity was just above the patient range. This case also had elevated CK,

ASAT, and ALAT levels. Both cases did not display clinical signs of Pompe disease and were diagnosed not to have Pompe disease. They illustrate the difficulty of GAA diagnosis solely based on biochemical assays.

Genetic analysis

DNA sequence analysis has become an alternative or supportive diagnostic tool in Pompe disease. We recently extended the Pompe mutation database, now termed the Pompe disease *GAA* variant database, with clinical information [www.pompevariantdatabase.nl] [1]. There are at least two reasons not to put DNA analysis first in line. First, two disease-associated *GAA* variants cannot always be identified by standard diagnostics, for example when the variant is located in an intron [2]. Second, it is not always known whether the variant is disease-associated, despite the large number of variants listed at www.pompevariantdatabase.nl and at the LOVD.

Our present studies focused on the performance of various enzymatic procedures for diagnosing Pompe disease. The pro's and con's of various genetic procedures are largely known and we mention them only briefly. Sequencing of only the *GAA* exons and adjacent regions bears the risk that disease-associated variants in the promoter sequences or deeper intronic sequences are missed. This may be approached using analysis at the mRNA level as shown by us previously [2,3]. Another limitation is the identification of variants of uncertain significance (VUS) necessitating the performance of an expression assay [4,5]. NGS procedures, developed for disease specific gene panels, are gaining field, but their application is expected to increase the number of VUSs. The great advantage of an enzymatic assay over DNA analysis is that the enzymatic assay captures in principle all disease-associated *GAA* variants in one. However, when it comes to carrier detection and genetic counseling in families with an index Pompe case, DNA analysis is essential since the activity ranges of carriers and healthy individuals show overlap [6].

Supplementary References

1. Nino MY, In 't Groen SLM, Bergsma AJ, van der Beek N, Kroos M, Hoogeveen-Westerveld M, et al. Extension of the Pompe mutation database by linking disease-associated variants to clinical severity. *Hum Mutat* 2019; **40:** 1954-1967.

2. Bergsma AJ, In 't Groen SL, Verheijen FW, van der Ploeg AT, Pijnappel W. From Cryptic Toward Canonical Pre-mRNA Splicing in Pompe Disease: a Pipeline for the Development of Antisense Oligonucleotides. *Mol Ther Nucleic Acids* 2016; **5:** e361.

3. Bergsma AJ, Kroos M, Hoogeveen-Westerveld M, Halley D, van der Ploeg AT, Pijnappel WW. Identification and characterization of aberrant GAA pre-mRNA splicing in pompe disease using a generic approach. *Hum Mutat* 2015; **36:** 57-68.

4. Kroos M, Pomponio RJ, van Vliet L, Palmer RE, Phipps M, Van der Helm R, et al. Update of the Pompe disease mutation database with 107 sequence variants and a format for severity rating. *Hum Mutat* 2008; **29:** E13-26.

5. Kroos M, Hoogeveen-Westerveld M, Michelakakis H, Pomponio R, Van der Ploeg A, Halley D, et al. Update of the pompe disease mutation database with 60 novel GAA sequence variants and additional studies on the functional effect of 34 previously reported variants. *Hum Mutat* 2012; **33:** 1161-1165.

6. Reuser A, Verheijen F, Kroos M, Okumiya T, Van Diggelen O, Van der Ploeg A, et al. Enzymatic and molecular strategies to diagnose Pompe disease. *Expert Opin Med Diagn* 2010; **4:** 79-89.

Supplementary Figure and Tables

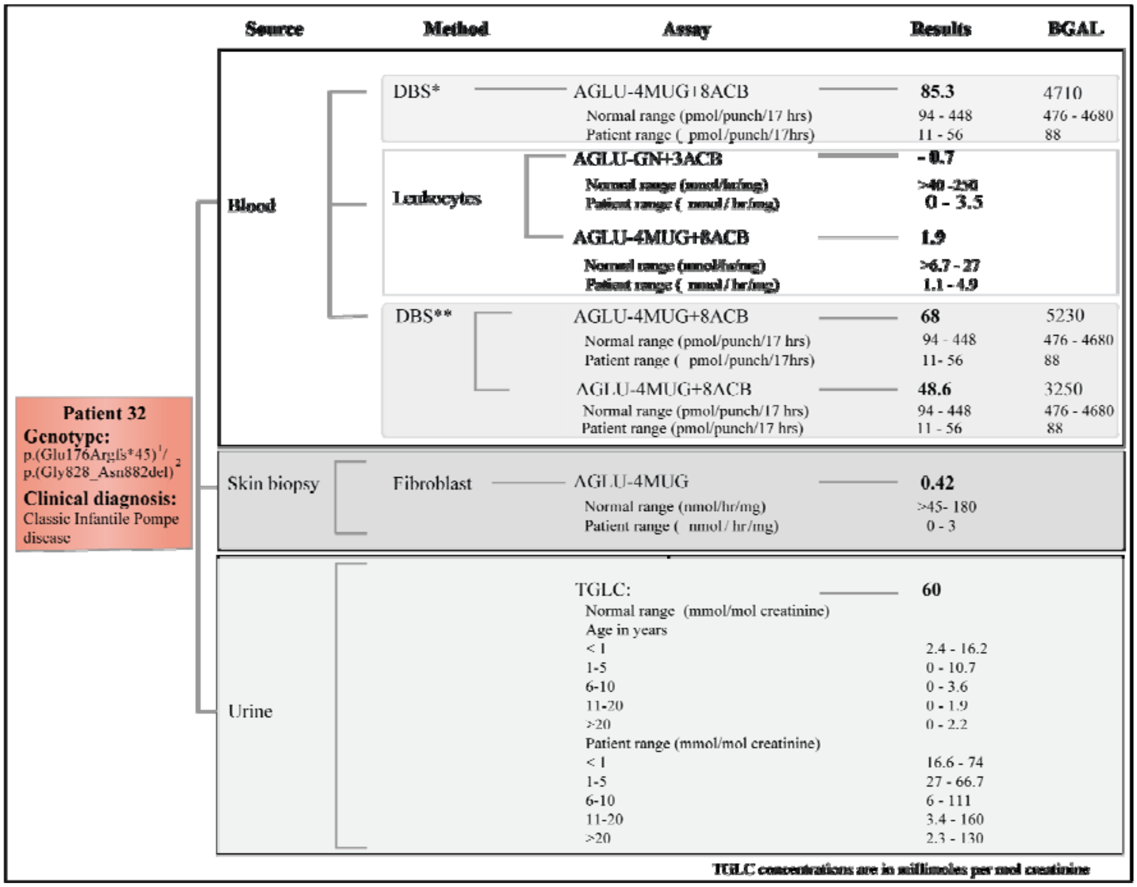

Supplementary Figure S1, Series of assays used for diagnosis of patient 32. DBSs, leukocytes and fibroblasts were analyzed as well as the Glc4 content of urine. [1] = pathogenic variant from the mother, [2] = pathogenic variant from the father, * a pilot study by DBS showed GAA activity in the gray zone, and BGAL activity higher than normal ranges. Technical replicates were therefore performed (**), as well as assays in leukocytes using glycogen or 4MUG (plus acarbose) as substrate and in fibroblast with 4MUG as substrate.

Supplementary Table S1. GAA enzymatic activities measured in all individuals.

Supplied as an .xlsx file
