# Supplementary material for: Enzymatic diagnosis of Pompe disease: lessons from 28 years of experience
Source: Eur J Hum Genet. 2020 Nov 8;29(3):434–46. doi: 10.1038/s41431-020-00752-2 (PMC7940434; doi:10.1038/s41431-020-00752-2)
Supplement: Supplementary file 2 — S1 [file 41431_2020_752_MOESM2_ESM.pdf]

Patient 32
Genotype:
p.(Glu176Argfs*45)[1]/
p.(Gly828_Asn882del)[2]
Clinical diagnosis:
Classic Infantile Pompe disease

Source	Method	Assay	Results	BGAL
Blood	DBS*	AGLU-4MUG+8ACB	**85.3**	4710
		Normal range (pmol/punch/17 hrs)	94 - 448	476 - 4680
		Patient range (pmol/punch/17hrs)	11 - 56	88
	Leukocytes	AGLU-GN+3ACB	**- 0.7**	
		Normal range (nmol/hr/mg)	>40 -250	
		Patient range (nmol / hr /mg)	0 - 3.5	
		AGLU-4MUG+8ACB	**1.9**	
		Normal range (nmol/hr/mg)	>6.7 - 27	
		Patient range (nmol / hr /mg)	1.1 - 4.9	
	DBS**	AGLU-4MUG+8ACB	**68**	5230
		Normal range (pmol/punch/17 hrs)	94 - 448	476 - 4680
		Patient range (pmol/punch/17hrs)	11- 56	88
		AGLU-4MUG+8ACB	**48.6**	3250
		Normal range (pmol/punch/17 hrs)	94 - 448	476 - 4680
		Patient range (pmol/punch/17 hrs)	11 - 56	88
Skin biopsy	Fibroblast	AGLU-4MUG	**0.42**	
		Normal range (nmol/hr/mg)	>45- 180	
		Patient range (nmol / hr /mg)	0 - 3	
Urine		TGLC:	**60**	
		Normal range (mmol/mol creatinine)		
		Age in years		
		< 1	2.4 - 16.2	
		1-5	0 - 10.7	
		6-10	0 - 3.6	
		11-20	0 - 1.9	
		>20	0 - 2.2	
		Patient range (mmol/mol creatinine)		
		< 1	16.6 - 74	
		1-5	27 - 66.7	
		6-10	6 - 111	
		11-20	3.4 - 160	
		>20	2.3 - 130	

TGLC concentrations are in millimoles per mol creatinine

Suppl Fig. S1, Niño et al. 2020
