# Supplementary material for: Enzymatic diagnosis of Pompe disease: lessons from 28 years of experience
Source: Eur J Hum Genet. 2020 Nov 8;29(3):434–46. doi: 10.1038/s41431-020-00752-2 (PMC7940434; doi:10.1038/s41431-020-00752-2)
Supplement: Supplementary file 3 — Table S1 [file 41431_2020_752_MOESM3_ESM.pdf]

Patient	Phenotype			Activity			
	Leu-Gly	Leu-4MU	Fibro	Leu-Gly	Leu-4MU	Fibro	# Assays
ID00376	Childhood	Childhood	Childhood	4.15	0.249	5.95	3
ID00998	Classic infantile	Classic Infantile	Classic Infantile	-1.41	0.597	0.3	3
ID00064	Adult	Adult	Adult	0.45	0.808	9.74	3
ID00734	Classic Infantile	Classic Infantile	Classic Infantile	-1.07	0.969	0.592	3
ID00008	Classic infantile	Classic Infantile		2.49	1.02		2
ID01004	Classic infantile	Classic Infantile		-0.505	1.03		2
ID00836	Childhood	Childhood	Childhood	0.848	1.16	11.6	3
ID00454	Classic Infantile	Classic Infantile	Classic Infantile	0.305	1.23	0.172	3
ID00867	Classic Infantile	Classic Infantile	Classic Infantile	1.64	1.31	0.412	3
ID01127	Adult	Adult	Adult	0.32	1.33	4.22	3
ID01260	Childhood	Childhood	Childhood	0.249	1.34	15.4	3
ID00465	Classic Infantile	Classic Infantile	Classic Infantile	1.24	1.35	0.436	3
ID00032	Adult	Adult		1.2	1.39		2
ID00457	Classic Infantile	Classic Infantile	Classic Infantile	-1.59	1.48	0.362	3
ID00870	Classic Infantile	Classic Infantile	Classic Infantile	-0.435	1.55	0.09	3
ID01276	Adult	Adult	Adult	1.35	1.57	5.38	3
ID01090	Adult	Adult	Adult	3.18	1.605	7.71	3
ID00346	Adult	Adult	Adult	1.83	1.615	6.16	3
ID00607	Classic Infantile	Classic Infantile	Classic Infantile	-0.108	1.645	0.327	3
ID00999	Adult	Adult		1.38	1.66		2
ID00034	Classic Infantile	Classic Infantile	Classic Infantile	5.04	1.69	0.239	3
ID00111	Adult	Adult		1.14	1.82		2
ID00472	Adult	Adult	Adult	1.76	1.84	11.2	3
ID00743	Classic Infantile	Classic Infantile		1.01	1.845		2
ID00963	Adult	Adult	Adult	1.5	1.86	7.59	3
ID00593	Classic Infantile	Classic Infantile	Classic Infantile	-0.71	1.92	0.415	3
ID00699	Adult	Adult	Adult	-0.449	1.92	0.541	3
ID00070	Adult	Adult		1.21	1.92		2
ID01086	Adult	Adult	Adult	2	1.935	19.2	3
ID01040	Childhood	Childhood	Childhood	6.71	1.98	2.31	3
ID00452	Adult	Adult	Adult	0.363	1.98	11.1	3
ID00790	Childhood	Childhood	Childhood	-0.53	2.03	0.359	3
ID00158	Classic infantile	Classic Infantile		0.42	2.06		2
ID00459	Adult	Adult	Adult	-0.361	2.08	13.5	3
ID00950	Adult	Adult		1.08	2.08		2
ID00458	Adult	Adult		-0.49	2.1		2
ID00669	Adult	Adult	Adult	2.23	2.12	12.5	3
ID00724	Classic Infantile	Classic Infantile		-0.212	2.15		2
ID00854	Childhood	Childhood	Childhood	1.8	2.17	8.72	3
ID01047	Classic Infantile	Classic Infantile		0.07	2.26		2
ID00589	Gray Zone	Unknown/Deficient		28.3	2.26		2
ID00056	Gray Zone	Unknown/Deficient	Gray zone	18.3	2.285	21.4	3
ID00237	Gray Zone	Unknown/Deficient		15	2.35		2
ID00340	Adult	Adult	Adult	1.83	2.385	6.31	3
ID01269	Adult	Adult		1.39	2.4		2
ID00661	Adult	Adult	Adult	0.588	2.46	13.9	3
ID00322	Adult	Adult	Adult	2.82	2.47	18.3	3

ID00252	Adult	Adult	Adult	2.6	2.475	8.3	3
ID00141	Adult	Adult	Adult	-0.591	2.48	0.56	3
ID00198	Adult	Adult		1.82	2.495		2
ID01143	Adult	Adult		1.91	2.6		2
ID01262	Adult	Adult	Adult	0.68	2.605	9.25	3
ID00254	Adult	Adult	Adult	2.38	2.61	8.77	3
ID00971	Adult	Adult	Adult	9.97	2.645	7.93	3
ID00653	Adult	Adult	Adult	2.14	2.66	11.2	3
ID00072	Gray Zone	Unknown/Deficient		20.9	2.74		2
ID01292	Adult	Adult		3.46	2.78		2
ID01293	Adult	Adult		5.35	2.81		2
ID01212	Adult	Adult	Adult	1.22	2.84	11.3	3
ID00668	Adult	Adult		0.929	2.84		2
ID00613	Adult	Adult		0.462	2.92		2
ID01125	Classic infantile	Classic Infantile	Classic Infantile	-0.672	2.96	0.251	3
ID01234	Adult	Adult	Adult	0.9	2.97	5.21	3
ID00519	Adult	Adult	Adult	0.91	2.975	12.7	3
ID00635	Adult	Adult	Adult	1.98	2.99	8.38	3
ID00961	Unknown/Deficient	Unknown/Deficient	Gray zone	9.61	3	22	3
ID00655	Adult	Adult		3.12	3		2
ID00527	Adult	Adult	Adult	-0.812	3.01	13.7	3
ID01039	Adult	Adult	Adult	0	3.04	17.2	3
ID00667	Adult	Adult	Adult	1.21	3.09	18.5	3
ID01136	Adult	Adult		2.68	3.15		2
ID00727	Adult	Adult		-1.36	3.19		2
ID00970	Adult	Adult		5.1	3.2		2
ID00127	Adult	Adult		1.11	3.21		2
ID01164	Adult	Adult		1.945	3.21		2
ID00308	Childhood	Childhood	Childhood	4.12	3.22	10.7	3
ID00372	Childhood	Childhood	Childhood	1.91	3.28	0.729	3
ID00251	Adult	Adult	Adult	4.22	3.3	5.95	3
ID00317	Gray zone	No Pompe/Deficient	Normal	22.1	3.32	46.3	3
ID01172	Adult	Adult	Adult	1.12	3.365	12.8	3
ID00853	Gray Zone	Unknown/Deficient	Gray zone	18.7	3.372	20.75	3
ID00957	Adult	Adult	Adult	2.3	3.38	10.3	3
ID00685	Adult	Adult		2.07	3.41		2
ID00658	Gray zone	No Pompe/Deficient	Normal	25.9	3.42	69.4	3
ID00381	Classic Infantile	Classic Infantile	Classic Infantile	21.8	3.46	0.219	3
ID01097	Adult	Adult	Adult	3.56	3.46	0.808	3
ID00356	Adult	Adult		-1.46	3.46		2
ID00485	Adult	Adult		1.36	3.46		2
ID00689	Adult	Adult	Adult	0.916	3.7	9.1	3
ID00850	Gray zone	Unknown/Deficient		28.5	3.845		2
ID00801	Adult	Adult	Adult	2.93	3.86	12.7	3
ID01034	Adult	Adult	Adult	-1.44	3.97	8.69	3
ID00923	Gray zone	Unknown/Deficient		18.9	3.98		2
ID00396	Adult	Adult		0.854	4		2
ID01201	Gray Zone	Unknown/Deficient		32.4	4.05		2
ID00235	Adult	Adult		3.19	4.08		2
ID00924	Classic Infantile	Classic Infantile	Classic Infantile	0.08	4.085	0.432	3

ID00120	Adult	Adult	Adult	1.04	4.1	11.7	3
ID00149	Adult	Adult		3.26	4.14		2
ID00219	Gray Zone	Unknown/Deficient		31.05	4.14		2
ID00288	Adult	Adult	Adult	1.76	4.27	11	3
ID00901	Childhood	Childhood	Childhood	4.29	4.3	13.7	3
ID00473	Childhood	Childhood	Childhood	1	4.39	0.386	3
ID00649	Adult	Adult	Adult	1.46	4.46	11.9	3
ID00039	Normal	Unknown/Deficient		47	4.48		2
ID00214	Gray Zone	Unknown/Deficient		35.6	4.51		2
ID00232		Unknown/Deficient			4.585		1
ID00326	Gray Zone	Unknown/Deficient		33.3	4.62		2
ID00225	Gray Zone	Unknown/Deficient		18	4.78		2
ID01152	Adult	Adult	Adult	1.85	4.79	14.8	3
ID00226	Adult	Adult		2.39	4.86		2
ID00146	Normal	Unknown/Deficient		45.7	4.86		2
ID01285	Adult	Adult	Adult	0.3	4.87	14.5	3
ID00145	Normal	Unknown/Deficient		46	4.87		2
ID00369	Normal	Gray zone		46.7	4.98		2
ID00133	Adult	Adult	Adult	2.18	4.99	15.7	3
ID00976	Gray Zone	Gray zone		20.3	5.06		2
ID00367	Gray zone	Gray zone	Asymptomatic/Deficient	31	5.07	15.25	3
ID00764	Gray zone	Gray zone		29.4	5.08		2
ID00888	Gray zone	Gray zone	Gray zone	19.2	5.11	39.1	3
ID00455	Normal	Gray zone		50.4	5.12		2
ID00139	Childhood	Childhood		0.365	5.14		2
ID00966	Normal	Gray zone		47.7	5.17		2
ID00778	Normal	Gray zone		44	5.18		2
ID00084	Normal	Gray zone		45.8	5.27		2
ID01020	Normal	Gray zone		46.1	5.33		2
ID00568	Unknown/Deficient	Gray zone	Gray zone	9.05	5.36	33.1	3
ID00855	Normal	Gray zone		40.1	5.37		2
ID00247	Normal	Gray zone		40.5	5.41		2
ID00714	Gray Zone	Gray zone		35	5.48		2
ID00453	Normal	Gray zone		44.8	5.48		2
ID00762	Normal	Gray zone		46.8	5.49		2
ID00320	Normal	Gray zone		47.3	5.49		2
ID00987	Gray Zone	Gray zone		38.2	5.53		2
ID01129	Gray Zone	Gray zone		28.4	5.54		2
ID00757	Normal	Gray zone		46.1	5.54		2
ID00220	Gray Zone	Gray zone		38.8	5.57		2
ID00523	Gray zone	Gray zone		35.4	5.58		2
ID00246	Normal	Gray zone		58.3	5.59		2
ID00323	Normal	Gray zone		61.2	5.635		2
ID00264	Gray Zone	Gray zone	Asymptomatic/Deficient	24	5.65	19.5	3
ID00956	Normal	Gray zone		47.2	5.66		2
ID00789	Normal	Gray zone		47.1	5.67		2
ID00880	Normal	Gray zone		41.3	5.68		2
ID01083	Normal	Gray zone		64.2	5.68		2
ID00716	Normal	Gray zone		55.6	5.84		2
ID00732	Normal	Gray zone		71.2	5.88		2

ID00641	Normal	Gray zone		52.4	5.89		2
ID00483	Normal	Gray zone		51.7	5.91		2
ID00960	Normal	Gray zone		46	5.94		2
ID00665	Normal	Gray zone		54.8	5.97		2
ID00468	Gray zone	Gray zone		21	5.99		2
ID00482	Normal	Gray zone		66.8	6.01		2
ID00730	Normal	Gray zone		63.8	6.02		2
ID00079	Gray zone	Gray zone	Gray zone	34.3	6.05	43.2	3
ID00973	Unknown/Deficient	Gray zone	Gray zone	2.2	6.06	25.9	3
ID00450	Normal	Gray zone		44	6.18		2
ID00298	Normal	Gray zone		62.2	6.26		2
ID01231	Normal	Gray zone		64.6	6.26		2
ID00279	Normal	Gray zone		74.6	6.31		2
ID00677	Normal	Gray zone		43.7	6.32		2
ID01013	Normal	Gray zone		44.7	6.32		2
ID00272	Normal	Gray zone		66.2	6.32		2
ID00494	Gray zone	Gray zone	Gray zone	36.6	6.46	41.9	3
ID01033	Gray zone	Gray zone		34.9	6.48		2
ID00884	No Pompe/Deficient	Gray zone	Normal	4.7	6.5	53.5	3
ID00774	Normal	Gray zone		57.7	6.53		2
ID00506	Normal	Gray zone		58.3	6.58		2
ID00353	Gray Zone	Gray zone		34.6	6.62		2
ID01005	Normal	Gray zone		44.8	6.64		2
ID01026	Normal	Gray zone		41.5	6.65		2
ID00807	Normal	Gray zone		48.3	6.66		2
ID00537	Normal	Gray zone		53.2	6.69		2
ID00142	Normal	Normal		45.7	6.73		2
ID00286	Normal	Normal		56	6.73		2
ID01294	Normal	Normal		62.2	6.76		2
ID01144	Normal	Normal		47.7	6.79		2
ID00207	Normal	Normal		49.6	6.81		2
ID00737	Normal	Normal		51.2	6.81		2
ID00648	Normal	Normal		48.7	6.87		2
ID00697	Normal	Normal		57.8	6.89		2
ID00121	Normal	Normal		62.9	6.92		2
ID00890	Normal	Normal		48.1	6.95		2
ID00441	Normal	Normal		57	6.97		2
ID00443	Normal	Normal		61.2	6.99		2
ID00078	Normal	Normal		53.2	7.03		2
ID00967	Normal	Normal		74.8	7.05		2
ID01206	Normal	Normal		42	7.12		2
ID00709	Normal	Normal		57.9	7.14		2
ID01257	No Pompe/Deficient	Normal	Normal	4.08	7.15	51.3	3
ID00074	Normal	Normal		58.3	7.15		2
ID00042	Normal	Normal		58.6	7.16		2
ID00926	Normal	Normal		65.9	7.16		2
ID00049	Normal	Normal		67.1	7.17		2
ID01085	Normal	Normal		57.4	7.18		2
ID00599	Normal	Normal		55.8	7.2		2
ID00261	Normal	Normal		57.9	7.2		2

ID00991	Normal	Normal		48.7	7.23		2
ID00584	Normal	Normal		54.6	7.23		2
ID00848	Normal	Normal		42.6	7.25		2
ID00464	Normal	Normal		57	7.26		2
ID00348	Normal	Normal		42.1	7.35		2
ID01275	Normal	Normal		56.5	7.39		2
ID01160	Normal	Normal		60.3	7.39		2
ID00894	Normal	Normal		58.4	7.41		2
ID00200	Normal	Normal		66.5	7.41		2
ID00962	Normal	Normal		73	7.46		2
ID01261	Normal	Normal		62.1	7.49		2
ID00129	Normal	Normal		67	7.49		2
ID01166	Normal	Normal		66	7.55		2
ID00752	Normal	Normal		71.2	7.57		2
ID00882	Normal	Normal		76.9	7.57		2
ID00989	Normal	Normal		45	7.58		2
ID01036	Normal	Normal		77.1	7.58		2
ID01131	Normal	Normal		78	7.63		2
ID01295	Normal	Normal		74.6	7.64		2
ID01094	Normal	Normal		71.2	7.66		2
ID01017	Normal	Normal	Unknown/Deficient	47.9	7.67	17	3
ID00954	Normal	Normal		69.9	7.67		2
ID00086	Normal	Normal		64.3	7.68		2
ID01169	Normal	Normal		47.4	7.73		2
ID00059	Normal	Normal		70.3	7.73		2
ID01248	Normal	Normal		63.2	7.74		2
ID00812	Normal	Normal		52.7	7.77		2
ID01280	Normal	Normal		54.5	7.79		2
ID00242	Normal	Normal		73.3	7.8		2
ID00202	Normal	Normal		53	7.82		2
ID01022	Normal	Normal		61.2	7.87		2
ID00979	Normal	Normal		57.4	7.88		2
ID00719	Normal	Normal		64.3	7.92		2
ID01072	Gray Zone	Normal	Normal	36.2	7.93	61.7	3
ID00913	Normal	Normal		59.5	7.93		2
ID00172	Gray zone	Normal		37.9	7.94		2
ID00841	Normal	Normal		79.3	7.97		2
ID00930	Gray Zone	Normal		37	8.03		2
ID00352	Normal	Normal		58.6	8.04		2
ID00750	Normal	Normal		66.3	8.04		2
ID00857	Normal	Normal		73.2	8.05		2
ID00978	Normal	Normal		54.4	8.07		2
ID00071	Normal	Normal		58.1	8.15		2
ID00637	Normal	Normal		85.5	8.18		2
ID00147	Normal	Normal		80.5	8.19		2
ID00331	Normal	Normal		57.1	8.2		2
ID00770	Normal	Normal		68	8.2		2
ID00018	Normal	Normal		94.7	8.22		2
ID01225	Normal	Normal		73.6	8.24		2
ID00088	Normal	Normal		76.5	8.26		2

ID01296	Normal	Normal		81.3	8.26		2
ID01246	Normal	Normal		73.8	8.3		2
ID01239	Normal	Normal		55.9	8.32		2
ID00370	Normal	Normal		68.8	8.37		2
ID00581	Normal	Normal		72.7	8.39		2
ID01211	Normal	Normal		87.6	8.39		2
ID00723	Gray Zone	Normal		39.65	8.42		2
ID01222	Normal	Normal		85.2	8.43		2
ID00424	Normal	Normal		72.5	8.47		2
ID00745	Gray Zone	Normal		27.5	8.48		2
ID01041	Normal	Normal		57	8.48		2
ID00873	Normal	Normal		73	8.48		2
ID00981	Normal	Normal	Normal	46.6	8.49	50.4	3
ID00496	Normal	Normal		65.8	8.49		2
ID00746	Normal	Normal		63.9	8.52		2
ID00670	Normal	Normal		64.7	8.52		2
ID00442	Normal	Normal		73.3	8.53		2
ID00183	Normal	Normal		69.7	8.55		2
ID00412	Normal	Normal		79.7	8.6		2
ID00578	Normal	Normal		82.3	8.62		2
ID00518	Normal	Normal		77.1	8.64		2
ID00371	Normal	Normal		83.6	8.65		2
ID00135	Normal	Normal		64	8.66		2
ID00840	Normal	Normal		107	8.66		2
ID00345	Normal	Normal		60.7	8.67		2
ID00834	Normal	Normal		64.8	8.79		2
ID00164	Normal	Normal		69.1	8.81		2
ID00490	Normal	Normal		70.9	8.82		2
ID00964	Normal	Normal		74.7	8.83		2
ID00280	Normal	Normal		79.3	8.86		2
ID01149	Normal	Normal		67.2	8.88		2
ID01077	Normal	Normal		80.4	8.89		2
ID00358	Normal	Normal		62	8.9		2
ID00082	Normal	Normal		80.6	8.91		2
ID01134	Normal	Normal		78.5	8.92		2
ID01297	Normal	Normal		101	8.97		2
ID00985	Normal	Normal		73.6	8.98		2
ID00889	Normal	Normal	Normal	72.6	9	78	3
ID00341	Normal	Normal		88.1	9.12		2
ID00965	Normal	Normal		86.6	9.14		2
ID00492	Normal	Normal		82.7	9.15		2
ID01198	Normal	Normal		63.5	9.16		2
ID00838	Normal	Normal		77.5	9.16		2
ID01213	Normal	Normal		40.7	9.17		2
ID00262	Normal	Normal		67.3	9.17		2
ID00969	Normal	Normal		90.2	9.19		2
ID00134	Gray zone	Normal	Gray zone	28.5	9.2	34.2	3
ID00765	Normal	Normal		71.2	9.24		2
ID00881	Normal	Normal		50.4	9.27		2
ID00010	Normal	Normal		55.9	9.27		2

ID00476	Normal	Normal	86.6	9.3		2
ID00780	Normal	Normal	78.4	9.32		2
ID00731	Normal	Normal	74.2	9.33		2
ID00852	Normal	Normal	91.4	9.33		2
ID00289	Normal	Normal	72.8	9.36		2
ID01084	Normal	Normal	70.7	9.37		2
ID01229	Normal	Normal	91.7	9.37		2
ID00510	Normal	Normal	62	9.4		2
ID00195	Normal	Normal	86	9.41		2
ID01137	Normal	Normal	61.6	9.42		2
ID00864	Normal	Normal	75.6	9.44		2
ID00291	Gray Zone	Normal	29.5	9.45		2
ID00379	Normal	Normal	54.9	9.45		2
ID00708	Normal	Normal	69	9.45		2
ID00783	Normal	Normal	65.7	9.49		2
ID00575	Normal	Normal	105	9.5		2
ID01266	Normal	Normal	67.3	9.52		2
ID01230	Normal	Normal	91.6	9.52		2
ID00351	Normal	Normal	79.3	9.67		2
ID01228	Normal	Normal	94.7	9.69		2
ID01244	Normal	Normal	71.2	9.76		2
ID00996	Normal	Normal	84.7	9.76		2
ID00350	Normal	Normal	70.9	9.77		2
ID00678	Normal	Normal	171	9.77		2
ID00060	Normal	Normal	67	9.81		2
ID00733	Normal	Normal	60.5	9.88		2
ID00623	Normal	Normal	98.5	9.88		2
ID01233	Normal	Normal	80.5	9.91		2
ID00389	Normal	Normal	72.9	9.93		2
ID00975	Normal	Normal	76.6	9.94		2
ID00240	Normal	Normal	79.1	9.95		2
ID00374	Normal	Normal	97.5	9.96		2
ID00736	Normal	Normal	72.9	9.98		2
ID00456	Normal	Normal	101	9.98		2
ID01278	Normal	Normal	82.7	10		2
ID00199	Normal	Normal	85.5	10		2
ID00205	Normal	Normal	100	10		2
ID00580	Normal	Normal	83.7	10.1		2
ID00271	Normal	Normal	83.8	10.1		2
ID00846	Normal	Normal	90.5	10.1		2
ID00051	Normal	Normal	92.9	10.1		2
ID00681	Normal	Normal	95.8	10.1		2
ID01216	Normal	Normal	73	10.2		2
ID01049	Normal	Normal	78	10.2		2
ID00092	Normal	Normal	82.5	10.2		2
ID00885	Normal	Normal	94.8	10.2		2
ID00469	Normal	Normal	96.4	10.2		2
ID00862	Normal	Normal	59.5	10.3		2
ID00842	Normal	Normal	77.8	10.3		2
ID00076	Normal	Normal	78.8	10.3		2

ID00617	Normal	Normal		79.2	10.3		2
ID00571	Normal	Normal		83.7	10.3		2
ID01120	Normal	Normal		85	10.3		2
ID00908	Normal	Normal		86.6	10.3		2
ID01074	Normal	Normal		94.8	10.3		2
ID00847	Normal	Normal		96.5	10.3		2
ID00608	Normal	Normal		99	10.3		2
ID01243	Normal	Normal		105	10.3		2
ID01283	Normal	Normal		75.9	10.4		2
ID01102	Normal	Normal		76.5	10.4		2
ID00974	Normal	Normal		82.5	10.4		2
ID00337	Normal	Normal		89	10.4		2
ID00215	Normal	Normal		96.3	10.4		2
ID01150	Normal	Normal		74.7	10.5		2
ID00123	Normal	Normal		77.6	10.5		2
ID00048	Normal	Normal		90.9	10.5		2
ID00886	Normal	Normal		94	10.5		2
ID00451	Normal	Normal		107	10.5		2
ID00612	Normal	Normal		122	10.5		2
ID01069	Normal	Normal		67.4	10.6		2
ID00652	Normal	Normal		67.6	10.6		2
ID01065	Normal	Normal		77.5	10.6		2
ID01281	Normal	Normal		77.7	10.6		2
ID01251	Normal	Normal		90	10.6		2
ID00640	Normal	Normal		92.9	10.6		2
ID01210	Normal	Normal		97.6	10.6		2
ID00980	Normal	Normal		113	10.6		2
ID00489	Normal	Normal		56.2	10.7		2
ID00066	Normal	Normal		95.4	10.7		2
ID00583	Normal	Normal		99.4	10.7		2
ID00360	Normal	Normal		101	10.7		2
ID01236	Normal	Normal		82.3	10.8		2
ID01298	Normal	Normal		82.5	10.8		2
ID00155	Normal	Normal		88.4	10.8		2
ID01117	Normal	Normal		92.3	10.8		2
ID00777	Normal	Normal		92.9	10.8		2
ID00004	Normal	Normal		93.7	10.8		2
ID00065	Normal	Normal		103	10.8		2
ID01162	No Pompe/Deficient	Normal	Normal	7.62	10.9	49.8	3
ID00333	Normal	Normal	Normal	88.7	10.9	72.7	3
ID00366	Normal	Normal		61.2	10.9		2
ID00760	Normal	Normal		68.9	10.9		2
ID00460	Normal	Normal		89.8	10.9		2
ID00087	Normal	Normal		92.4	10.9		2
ID01073	Normal	Normal		95.3	10.9		2
ID00634	Normal	Normal		87.6	11		2
ID00470	Normal	Normal		96.6	11		2
ID00321	Normal	Normal		111	11		2
ID00951	Normal	Normal		111	11		2
ID00509	No Pompe/Deficient	Normal	Normal	4.35	11.1	86.9	3

ID00259	Normal	Normal		77.4	11.1		2
ID00968	Normal	Normal		86	11.1		2
ID00628	Normal	Normal		89.7	11.1		2
ID00163	Normal	Normal		95.9	11.1		2
ID00713	Normal	Normal		101	11.1		2
ID00776	Normal	Normal		103	11.1		2
ID00983	Normal	Normal		107	11.1		2
ID00335	Normal	Normal		125	11.1		2
ID01130	Normal	Normal		82.1	11.2		2
ID01214	Normal	Normal		91.5	11.2		2
ID00700	Normal	Normal		95.4	11.2		2
ID00090	Normal	Normal		86.2	11.3		2
ID00533	Normal	Normal		93.1	11.3		2
ID01221	Normal	Normal		95.3	11.3		2
ID00449	Normal	Normal		79.2	11.4		2
ID01114	Normal	Normal		89.7	11.4		2
ID00138	Normal	Normal		101	11.4		2
ID00793	Normal	Normal		81.4	11.5		2
ID00540	Normal	Normal		89.3	11.5		2
ID01237	Normal	Normal		94.8	11.5		2
ID01126	Normal	Normal		95.7	11.5		2
ID00067	Normal	Normal		115	11.5		2
ID00753	Normal	Normal		48.2	11.6		2
ID00526	Normal	Normal		83.5	11.6		2
ID00803	Normal	Normal		87.8	11.6		2
ID00355	Normal	Normal		88.4	11.6		2
ID01158	Normal	Normal		88.8	11.6		2
ID00997	Normal	Normal		102	11.6		2
ID00301	Normal	Normal		103	11.6		2
ID00576	Normal	Normal		115	11.6		2
ID00217	Normal	Normal		118	11.6		2
ID00328	Normal	Normal		85.1	11.7		2
ID01093	Normal	Normal		96.1	11.7		2
ID01135	Normal	Normal		113	11.7		2
ID00715	Normal	Normal		85	11.8		2
ID00475	Normal	Normal		116	11.8		2
ID01182	Normal	Normal		119	11.8		2
ID00314	Normal	Normal		120	11.8		2
ID00193	Normal	Normal	Normal	89.7	11.9	155	3
ID00124	Normal	Normal		67.9	11.9		2
ID00988	Normal	Normal		84	11.9		2
ID00596	Normal	Normal		92.2	11.9		2
ID00895	Normal	Normal		94.5	11.9		2
ID00294	Normal	Normal		96.5	11.9		2
ID00043	Normal	Normal		97.8	11.9		2
ID00738	Normal	Normal		101	11.9		2
ID00910	Normal	Normal		101	11.9		2
ID00327	Normal	Normal		109	11.9		2
ID00380	Normal	Normal		110	11.9		2
ID00728	Normal	Normal		130	11.9		2

ID01161	Normal	Normal		84.8	12		2
ID00038	Normal	Normal		89.6	12		2
ID01208	Normal	Normal		97.8	12		2
ID00466	Normal	Normal		114	12		2
ID00602	Normal	Normal		133	12		2
ID00045	Normal	Normal		93.2	12.1		2
ID01116	Normal	Normal		93.9	12.1		2
ID00017	Normal	Normal		109	12.1		2
ID01081	Normal	Normal		116	12.1		2
ID00815	Normal	Normal		77.6	12.2		2
ID00845	Normal	Normal		86.7	12.2		2
ID00165	Normal	Normal		90.1	12.2		2
ID00782	Normal	Normal		90.6	12.2		2
ID00875	Normal	Normal		99.8	12.2		2
ID01245	Normal	Normal		126	12.2		2
ID00196	Normal	Normal		76.1	12.3		2
ID01196	Normal	Normal		87.7	12.3		2
ID00565	Normal	Normal		99.3	12.3		2
ID00154	Normal	Normal		101	12.3		2
ID00907	Normal	Normal		94.2	12.4		2
ID00871	Normal	Normal		111	12.4		2
ID00203	Normal	Normal		113	12.4		2
ID00710	Normal	Normal		116	12.4		2
ID00477	Normal	Normal		109	12.5		2
ID00630	Normal	Normal		112	12.5		2
ID00982	Normal	Normal		115	12.5		2
ID00712	Normal	Normal		123	12.5		2
ID00493	Normal	Normal		75.1	12.6		2
ID01217	Normal	Normal		118	12.6		2
ID00690	Normal	Normal		147	12.6		2
ID00830	Normal	Normal		86.5	12.7		2
ID00769	Normal	Normal		86.7	12.7		2
ID00290	Normal	Normal		87	12.7		2
ID01223	Normal	Normal		105	12.7		2
ID00310	Normal	Normal		117	12.7		2
ID00312	Normal	Normal		126	12.7		2
ID00265	Normal	Normal		132	12.7		2
ID01165	Normal	Normal		106	12.8		2
ID01071	Normal	Normal	Normal	155	12.9	47.9	3
ID00503	Normal	Normal		83.1	12.9		2
ID00990	Normal	Normal		93.5	12.9		2
ID00717	Normal	Normal		98.4	12.9		2
ID00053	Normal	Normal		118	12.9		2
ID00171	Normal	Normal		70.2	13		2
ID01016	Normal	Normal		89.7	13		2
ID00461	Normal	Normal		112	13		2
ID00994	Normal	Normal		126	13		2
ID01299	Normal	Normal		142	13		2
ID01124	Normal	Normal		87.9	13.1		2
ID00874	Normal	Normal		88.4	13.1		2

ID00068	Normal	Normal	133	13.1		2
ID00486	Normal	Normal	100	13.2		2
ID00551	Normal	Normal	113	13.2		2
ID00860	Normal	Normal	116	13.2		2
ID00311	Normal	Normal	133	13.2		2
ID00040	Normal	Normal	156	13.2		2
ID00083	Normal	Normal	104	13.3		2
ID00795	Normal	Normal	112	13.3		2
ID00499	Normal	Normal	122	13.3		2
ID01291	Normal	Normal	124	13.3		2
ID01300	Normal	Normal	132	13.3		2
ID00843	Normal	Normal	143	13.3		2
ID00377	Normal	Normal	99.5	13.4		2
ID01123	Normal	Normal	117	13.4		2
ID00878	Normal	Normal	122	13.4		2
ID00644	Normal	Normal	126	13.4		2
ID00244	Normal	Normal	129	13.4		2
ID00675	Normal	Normal	85.7	13.5		2
ID00791	Normal	Normal	91.4	13.5		2
ID00329	Normal	Normal	143	13.5		2
ID01141	Normal	Normal	92.5	13.6		2
ID00248	Normal	Normal	107	13.6		2
ID00197	Normal	Normal	126	13.6		2
ID00047	Normal	Normal	130	13.6		2
ID00869	Normal	Normal	150	13.6		2
ID00243	Normal	Normal	102	13.7		2
ID01092	Normal	Normal	103	13.7		2
ID00522	Normal	Normal	110	13.7		2
ID00758	Normal	Normal	112	13.7		2
ID00680	Normal	Normal	116	13.7		2
ID01224	Normal	Normal	88.2	13.8		2
ID01252	Normal	Normal	109	13.8		2
ID01183	Normal	Normal	129	13.8		2
ID00859	Normal	Normal	108	13.9		2
ID01007	Normal	Normal	111	13.9		2
ID00266	Normal	Normal	117	13.9		2
ID00249	Normal	Normal	120	14		2
ID00835	Normal	Normal	147	14		2
ID00958	Normal	Normal	114	14.1		2
ID00062	Normal	Normal	136	14.1		2
ID01218	Normal	Normal	147	14.1		2
ID01095	Normal	Normal	94.9	14.2		2
ID01121	Normal	Normal	108	14.2		2
ID00361	Normal	Normal	119	14.2		2
ID00338	Normal	Normal	121	14.2		2
ID00792	Normal	Normal	111	14.3		2
ID01037	Normal	Normal	102	14.4		2
ID00742	Normal	Normal	122	14.4		2
ID01259	Normal	Normal	130	14.4		2
ID00416	Normal	Normal	135	14.4		2

ID00619	Normal	Normal	95.9	14.5		2
ID00204	Normal	Normal	166	14.5		2
ID00439	Normal	Normal	112	14.6		2
ID00707	Normal	Normal	129	14.6		2
ID00507	Normal	Normal	132	14.6		2
ID00721	Normal	Normal	143	14.6		2
ID01226	Normal	Normal	146	14.6		2
ID00170	Normal	Normal	114	14.7		2
ID00334	Normal	Normal	145	14.7		2
ID01099	Normal	Normal	151	14.7		2
ID00054	Normal	Normal	166	14.7		2
ID01173	Normal	Normal	109	14.8		2
ID00176	Normal	Normal	129	14.8		2
ID00349	Normal	Normal	145	14.8		2
ID00829	Normal	Normal	116	14.9		2
ID00610	Normal	Normal	123	14.9		2
ID01267	Normal	Normal	140	14.9		2
ID00342	Normal	Normal	201	14.9		2
ID00392	Normal	Normal	101	15		2
ID00354	Normal	Normal	107	15		2
ID00028	Normal	Normal	108	15		2
ID00137	Normal	Normal	118	15		2
ID01080	Normal	Normal	98.9	15.1		2
ID00297	Normal	Normal	139	15.1		2
ID00332	Normal	Normal	156	15.1		2
ID00319	Normal	Normal	108	15.2		2
ID00931	Normal	Normal	122	15.2		2
ID01000	Normal	Normal	137	15.2		2
ID00019	Normal	Normal	138	15.2		2
ID00763	Normal	Normal	140	15.2		2
ID00586	Normal	Normal	220	15.2		2
ID01171	Normal	Normal	135	15.3		2
ID00735	Normal	Normal	167	15.3		2
ID00175	Normal	Normal	80.4	15.4		2
ID00598	Normal	Normal	140	15.4		2
ID00603	Normal	Normal	146	15.4		2
ID00221	Normal	Normal	124	15.5		2
ID00953	Normal	Normal	144	15.5		2
ID00992	Normal	Normal	156	15.5		2
ID00577	Normal	Normal	158	15.5		2
ID00897	Normal	Normal	122	15.6		2
ID01038	Normal	Normal	131	15.6		2
ID00347	Normal	Normal	137	15.6		2
ID00069	Normal	Normal	139	15.6		2
ID00098	Normal	Normal	147	15.6		2
ID00609	Normal	Normal	125	15.7		2
ID00718	Normal	Normal	157	15.7		2
ID01079	Normal	Normal	123	15.8		2
ID01128	Normal	Normal	127	15.8		2
ID01235	Normal	Normal	130	15.8		2

ID00606	Normal	Normal	148	15.8		2
ID00570	Normal	Normal	153	15.8		2
ID00554	Normal	Normal	109	15.9		2
ID01070	Normal	Normal	142	15.9		2
ID00213	Normal	Normal	168	15.9		2
ID00767	Normal	Normal	139	16		2
ID00130	Normal	Normal	148	16		2
ID00616	Normal	Normal	160	16		2
ID01023	Normal	Normal	111	16.1		2
ID01009	Normal	Normal	116	16.1		2
ID00029	Normal	Normal	143	16.1		2
ID00343	Normal	Normal	113	16.2		2
ID00646	Normal	Normal	121	16.2		2
ID00909	Normal	Normal	108	16.3		2
ID00614	Normal	Normal	122	16.3		2
ID00739	Normal	Normal	129	16.3		2
ID00230	Normal	Normal	127	16.4		2
ID00747	Normal	Normal	132	16.4		2
ID00892	Normal	Normal	152	16.4		2
ID01075	Normal	Normal	161	16.4		2
ID01220	Normal	Normal	147	16.5		2
ID00627	Normal	Normal	123	16.6		2
ID00704	Normal	Normal	160	16.6		2
ID00515	Normal	Normal	131	16.7		2
ID00756	Normal	Normal	143	16.7		2
ID00899	Normal	Normal	157	16.7		2
ID00136	Normal	Normal	173	16.7		2
ID00304	Normal	Normal	121	16.8		2
ID01167	Normal	Normal	140	16.9		2
ID01051	Normal	Normal	167	16.9		2
ID00044	Normal	Normal	148	17		2
ID00585	Normal	Normal	130	17.1		2
ID00629	Normal	Normal	151	17.1		2
ID00779	Normal	Normal	163	17.1		2
ID01053	Normal	Normal	181	17.1		2
ID00041	Normal	Normal	124	17.2		2
ID01019	Normal	Normal	130	17.2		2
ID00189	Normal	Normal	140	17.2		2
ID00330	Normal	Normal	155	17.3		2
ID00594	Normal	Normal	175	17.3		2
ID01100	Normal	Normal	141	17.4		2
ID00174	Normal	Normal	113	17.5		2
ID01103	Normal	Normal	124	17.5		2
ID00672	Normal	Normal	127	17.5		2
ID00015	Normal	Normal	154	17.5		2
ID00597	Normal	Normal	177	17.5		2
ID01088	Normal	Normal	191	17.5		2
ID00579	Normal	Normal	166	17.7		2
ID01209	Normal	Normal	180	17.75		2
ID00276	Normal	Normal	154	17.8		2

ID00588	Normal	Normal	139	17.9		2
ID00073	Normal	Normal	105	18		2
ID01063	Normal	Normal	136	18		2
ID00114	Normal	Normal	154	18		2
ID00839	Normal	Normal	208	18		2
ID00844	Normal	Normal	164	18.1		2
ID00977	Normal	Normal	198	18.1		2
ID00487	Normal	Normal	127	18.3		2
ID00748	Normal	Normal	141	18.4		2
ID00385	Normal	Normal	149	18.4		2
ID00036	Normal	Normal	157	18.4		2
ID00701	Normal	Normal	138	18.5		2
ID01250	Normal	Normal	173	18.6		2
ID01091	Normal	Normal	197	18.6		2
ID00663	Normal	Normal	146	18.7		2
ID01279	Normal	Normal	146	18.8		2
ID01018	Normal	Normal	148	18.8		2
ID00097	Normal	Normal	249	18.8		2
ID00572	Above normal range	Normal	254	18.8		2
ID00711	Normal	Normal	147	18.9		2
ID01268	Normal	Normal	153	18.9		2
ID01254	Normal	Normal	163	18.9		2
ID00161	Normal	Normal	137	19		2
ID00103	Normal	Normal	190	19.3		2
ID00806	Normal	Normal	128	19.4		2
ID00508	Normal	Normal	146	19.4		2
ID00534	Normal	Normal	147	19.4		2
ID01110	Normal	Normal	179	19.4		2
ID00592	Normal	Normal	213	19.5		2
ID00865	Normal	Normal	141	19.6		2
ID00148	Normal	Normal	152	19.6		2
ID00387	Normal	Normal	152	19.6		2
ID00091	Normal	Normal	189	19.8		2
ID00156	Normal	Normal	154	19.9		2
ID00151	Normal	Normal	146	20		2
ID01115	Normal	Normal	181	20		2
ID01265	Normal	Normal	184	20		2
ID00152	Normal	Normal	186	20		2
ID00751	Normal	Normal	171	20.2		2
ID01301	Normal	Normal	209	20.2		2
ID00273	Normal	Normal	150	20.25		2
ID00013	Normal	Normal	148	20.4		2
ID00382	Normal	Normal	164	20.4		2
ID00471	Normal	Normal	158	20.6		2
ID00375	Normal	Normal	192	20.6		2
ID01011	Normal	Normal	188	20.9		2
ID00131	Normal	Normal	226	21		2
ID00773	Normal	Normal	157	21.2		2
ID00132	Normal	Normal	146	21.3		2
ID00324	Normal	Normal	153	21.5		2

ID01105	Normal	Normal		183	21.5		2
ID01241	Normal	Normal		188	21.5		2
ID00357	Normal	Normal		163	21.6		2
ID00687	Normal	Normal		201	21.8		2
ID00440	Normal	Normal		219	22		2
ID00546	Normal	Normal		165	22.1		2
ID00955	Normal	Normal		202	22.3		2
ID00046	Normal	Normal		213	22.3		2
ID00274	Normal	Normal		175	22.6		2
ID00140	Normal	Normal		184	23		2
ID01062	Normal	Normal		216	23.1		2
ID00505	Normal	Normal		214	23.3		2
ID01184	Normal	Normal		242	23.4		2
ID01277	Normal	Normal		201	24.2		2
ID00030	Normal	Normal		204	24.2		2
ID00143	Normal	Normal		168	24.3		2
ID00208	Normal	Normal		250	24.3		2
ID01302	Normal	Normal		192	24.4		2
ID01181	Normal	Normal		209	24.5		2
ID01227	Normal	Normal		246	24.5		2
ID00227	Normal	Normal		223	25.7		2
ID00706	Normal	Normal		238	25.9		2
ID00408	Normal	Normal		228	26.2		2
ID00362	Normal	Normal		220	26.5		2
ID00125	Normal	Above normal range		170	27.3		2
ID00500	Normal	Above normal range		232	27.4		2
ID01207	Above normal range	Above normal range		297	27.4		2
ID00622	Above normal range	Above normal range		252	31.3		2
ID00488	Above normal range	Above normal range		284	31.6		2
ID00368			Classic Infantile			-0.15	1
ID01327			Classic Infantile			0.03	1
ID00939			Classic Infantile			0.156	1
ID00325			Classic Infantile			0.178	1
ID01328			Classic Infantile			0.18	1
ID01329			Classic Infantile			0.18	1
ID01330			Classic Infantile			0.187	1
ID01331			Classic Infantile			0.22	1
ID01057	Classic Infantile		Classic Infantile	0.063		0.221	2
ID00995			Classic Infantile			0.227	1
ID00256			Classic Infantile			0.23	1
ID01332			Classic Infantile			0.235	1
ID01176	Classic Infantile		Classic Infantile	1.17		0.238	2
ID01333			Classic Infantile			0.24	1
ID01334			Classic Infantile			0.265	1
ID01335			Classic Infantile			0.27	1
ID01336			Classic Infantile			0.285	1
ID00633			Classic Infantile			0.302	1
ID00905	Childhood		Childhood	-0.225		0.305	2
ID01337			Classic Infantile			0.31	1
ID01338			Classic Infantile			0.33	1

ID01339		Classic Infantile		0.335	1
ID01340		Classic Infantile		0.337	1
ID01341		Classic Infantile		0.34	1
ID01342		Classic Infantile		0.34	1
ID01343		Classic Infantile		0.34	1
ID01344		Classic Infantile		0.34	1
ID00517	Classic Infantile	Classic Infantile	0	0.35	2
ID01345		Unknown/Deficient		0.371	1
ID01346		Classic Infantile		0.4	1
ID01347		Classic Infantile		0.42	1
ID01348		Classic Infantile		0.426	1
ID01349		Classic Infantile		0.43	1
ID01350		Classic Infantile		0.43	1
ID01351		Classic Infantile		0.43	1
ID01352		Classic Infantile		0.44	1
ID00642		Classic Infantile		0.448	1
ID01353		Classic Infantile		0.45	1
ID00268		Classic Infantile		0.465	1
ID01354		Classic Infantile		0.47	1
ID01355		Classic Infantile		0.47	1
ID01356		Classic Infantile		0.475	1
ID01357		Classic Infantile		0.483	1
ID00239		Classic Infantile		0.51	1
ID00726		Classic Infantile		0.51	1
ID00972		Classic Infantile		0.51	1
ID01358		Classic Infantile		0.51	1
ID01089		Classic Infantile		0.52	1
ID01359		Classic Infantile		0.52	1
ID01360		Classic Infantile		0.53	1
ID00725		Classic Infantile		0.57	1
ID01361		Classic Infantile		0.576	1
ID01362		Classic Infantile		0.59	1
ID01363		Unknown/Deficient		0.66	1
ID01364		Classic Infantile		0.68	1
ID01365		Classic Infantile		0.69	1
ID01366		Classic Infantile		0.7	1
ID01367		Classic Infantile		0.73	1
ID01368		Classic Infantile		0.752	1
ID01369		Classic Infantile		0.765	1
ID01370		Classic Infantile		0.825	1
ID01371		Classic Infantile		0.863	1
ID01372		Classic Infantile		0.9	1
ID01373		Unknown/Deficient		1.14	1
ID01374		Classic Infantile		1.17	1
ID01375		Classic Infantile		1.63	1
ID01031		Childhood		1.72	1
ID00080	Childhood	Childhood	1.92	2.07	2
ID01376		Classic Infantile		2.2	1
ID00514		Childhood		2.33	1
ID01377		Classic Infantile		2.49	1

ID00398	Childhood	Childhood	-0.604		4.15	2
ID01082		Childhood			4.61	1
ID01378		Adult			6.04	1
ID00415		Childhood			6.17	1
ID01379		Asymptomatic/Deficient			7.25	1
ID00110	Adult	Adult	1.2		7.33	2
ID00525	Adult	Adult	3.27		7.41	2
ID00914		Adult			7.57	1
ID00115	Childhood	Childhood	0.71		7.79	2
ID00887	Adult	Adult	2.84		7.85	2
ID00055	Adult	Adult	6.11		8.01	2
ID01151	Adult	Adult	2.78		8.27	2
ID01380		Adult			8.28	1
ID00122	Adult	Adult	-0.342		8.35	2
ID01381		Unknown/Deficient			8.35	1
ID00904	Childhood	Childhood	2.96		8.38	2
ID01247		Adult			8.485	1
ID01382		Classic Infantile			8.51	1
ID00766		Childhood			8.57	1
ID01174		Childhood			8.87	1
ID00052		Childhood			9.1	1
ID01187	Adult	Adult	3.47		9.22	2
ID00384	Adult	Adult	2.88		9.39	2
ID01112	Adult	Adult	2.26		9.4	2
ID00050		Adult			9.76	1
ID01282	Adult	Adult	1.82		10	2
ID00478	Adult	Adult	1.3		10.02	2
ID01383		Adult			10.3	1
ID00813		Adult			10.4	1
ID00359		Adult			10.45	1
ID00820	Adult	Adult	4.07		10.5	2
ID00684	Adult	Adult	2.23		10.6	2
ID01384		Asymptomatic/Deficient			10.7	1
ID00108	Adult	Adult	1.59		10.8	2
ID01159	Adult	Adult	2.38		10.9	2
ID00497	Adult	Adult	-0.559		10.953	2
ID01385		Adult			11.05	1
ID00636	Adult	Adult	0		11.1	2
ID00787	Adult	Adult	7.25		11.1	2
ID01157	Adult	Adult	2.23		11.3	2
ID00639	Adult	Adult	2.67		11.6	2
ID00096	Adult	Adult	2.75		11.75	2
ID00373	Adult	Adult	4.02		11.8	2
ID00927	Adult	Adult	4.19		11.8	2
ID00810	Adult	Adult	0.23		12.2	2
ID00643	Childhood	Childhood	1.27		12.2	2
ID00263		Adult			12.2	1
ID00474	Adult	Adult	3.765		12.25	2
ID00542	Adult	Adult	2.3		12.6	2
ID01386		Adult			12.7	1

ID00660	Adult	Adult	3.86		12.8	2
ID01387		Unknown/Deficient			13	1
ID00109		Adult			13.1	1
ID00378	Childhood	Childhood	5.08		13.2	2
ID01388		Adult			13.2	1
ID00113	Adult	Adult	0.74		13.3	2
ID00896	Adult	Adult	0.59		13.35	2
ID00945	Adult	Adult	3.57		13.5	2
ID01274	Adult	Adult	4.7		13.5	2
ID00898	Adult	Adult	4.39		13.7	2
ID01118	Adult	Adult	1.51		14	2
ID00682	Adult	Adult	-0.61		14.1	2
ID00021	Adult	Adult	0.126		14.6	2
ID01032	Adult	Adult	2.67		14.66	2
ID01028	Adult	Adult	1.5		14.7	2
ID01146		Adult			14.8	1
ID01012	Adult	Adult	-1.06		14.9	2
ID00281	Adult	Adult	0.93		15	2
ID00012	Adult	Adult	1.22		15	2
ID00564	Adult	Adult	1.9		15.2	2
ID00501	Adult	Adult	2.9		15.3	2
ID00427	Adult	Adult	2.48		15.6	2
ID01030		Childhood			15.9	1
ID00528	Adult	Adult	0.137		16.1	2
ID01389		Adult			16.1	1
ID00520		Adult			16.2	1
ID01253		Adult			16.3	1
ID01284	Adult	Adult	1.46		16.9	2
ID00925		Adult			16.9	1
ID00391		Adult			17.2	1
ID01390		Adult			17.4	1
ID00804	Adult	Adult	0		17.7	2
ID01006	Childhood	Childhood	6.87		17.9	2
ID00409	Adult	Adult	3.71		18	2
ID00567	Adult	Adult	0.218		18.2	2
ID00548		Adult			18.3	1
ID00772		Unknown/Deficient			18.3	1
ID01391		Asymptomatic/Deficient			19.5	1
ID00771	Adult	Adult	2.39		19.85	2
ID01392		Asymptomatic/Deficient			19.9	1
ID01113		Gray zone			20.1	1
ID00722		Gray zone			20.75	1
ID01393		Gray zone			20.9	1
ID01394		Gray zone			21.1	1
ID01395		Gray zone			21.55	1
ID01396		Gray zone			22.2	1
ID00101		Gray zone			22.8	1
ID01397		Gray zone			24.4	1
ID00798		Gray zone			24.7	1
ID01398		Gray zone			24.7	1

ID01399		Gray zone		25	1
ID01400		Gray zone		25.1	1
ID01401		Gray zone		25.6	1
ID01402		Gray zone		25.9	1
ID01403		Gray zone		26.7	1
ID01404		Gray zone		28.1	1
ID00768		Gray zone		28.6	1
ID01405		Gray zone		29.1	1
ID01406		Gray zone		29.5	1
ID01010		Gray zone		30.2	1
ID01407		Gray zone		30.25	1
ID01408		Gray zone		31	1
ID00395		Gray zone		31.6	1
ID00075		Gray zone		33	1
ID00411	No Pompe/Deficient	Gray zone	5.71	33.3	2
ID01409		Gray zone		33.4	1
ID01410		Gray zone		33.5	1
ID01411		Gray zone		33.9	1
ID01412		Gray zone		34.6	1
ID00058		Gray zone		35.7	1
ID01413		Gray zone		36	1
ID01414		Gray zone		36.4	1
ID01415		Gray zone		36.8	1
ID00861		Gray zone		36.9	1
ID00245		Gray zone		37.6	1
ID01416		Gray zone		38.3	1
ID00754		Gray zone		38.4	1
ID00624	Gray zone	Gray zone	15.6	38.6	2
ID01417		Gray zone		39.1	1
ID00275		Gray zone		39.3	1
ID01418		Gray zone		40	1
ID01419		Gray zone		40.4	1
ID01420		Gray zone		40.5	1
ID01421		Gray zone		41.3	1
ID00984		Gray zone		42.55	1
ID01272		Gray zone		42.9	1
ID00936		Gray zone		43.3	1
ID01422		Gray zone		44.1	1
ID00224		Gray zone		44.3	1
ID01423		Gray zone		44.3	1
ID00902		Gray zone		44.5	1
ID01193		Gray zone		44.9	1
ID01424		Normal		45.3	1
ID01425		Normal		46.2	1
ID01426		Normal		46.4	1
ID00363		Normal		47.1	1
ID01427		Normal		47.1	1
ID01428		Normal		47.1	1
ID00495		Normal		47.6	1
ID01429		Normal		47.6	1

ID01430		Normal		47.9	1
ID00480		Normal		48.2	1
ID00085		Normal		48.6	1
ID01431		Normal		48.6	1
ID01432		Normal		48.7	1
ID01433		Normal		48.7	1
ID01434		Normal		48.8	1
ID01435		Normal		49	1
ID01436		Normal		49.3	1
ID00825		Normal		49.5	1
ID01437		Normal		49.75	1
ID00582		Normal		49.8	1
ID00651		Normal		50.3	1
ID01438		Normal		50.4	1
ID01439		Normal		50.4	1
ID00467		Normal		50.7	1
ID01140	No Pompe/Deficient	Normal	4.12	51	2
ID00126		Normal		51.2	1
ID01440		Normal		51.4	1
ID01441		Normal		51.4	1
ID01442		Normal		51.5	1
ID01443		Normal		51.6	1
ID01444		Normal		52.4	1
ID01145	No Pompe/Deficient	Normal	6.58	53.1	2
ID01445		Normal		54	1
ID01446		Normal		54.2	1
ID01447		Normal		54.2	1
ID01448		Normal		54.3	1
ID01449		Normal		54.3	1
ID01450		Normal		54.6	1
ID00744		Normal		54.9	1
ID01451		Normal		54.9	1
ID01452		Normal		55.2	1
ID01453		Normal		55.2	1
ID01454		Normal		55.3	1
ID01455		Normal		55.4	1
ID01456		Normal		55.65	1
ID01457		Normal		56.6	1
ID01458		Normal		57.2	1
ID01459		Normal		57.6	1
ID00206		Normal		57.7	1
ID01460		Normal		57.8	1
ID01461		Normal		58	1
ID01462		Normal		58.3	1
ID01463		Normal		58.5	1
ID01464		Normal		58.7	1
ID01465		Normal		59.3	1
ID01466		Normal		59.8	1
ID01467		Normal		60.1	1
ID01468		Normal		60.4	1

ID01111	Normal		60.5	1
ID01469	Normal		60.5	1
ID01470	Normal		60.7	1
ID01471	Normal		61	1
ID01472	Normal		61.1	1
ID01473	Normal		61.1	1
ID01474	Normal		61.2	1
ID00253	Normal		61.4	1
ID01475	Normal		61.8	1
ID01476	Normal		61.9	1
ID01477	Normal		62.25	1
ID01478	Normal		62.3	1
ID01479	Normal		62.5	1
ID00393	Normal		62.7	1
ID01480	Normal		62.85	1
ID00740	Normal		63.1	1
ID00879	Normal		63.4	1
ID01068	Normal		63.9	1
ID01481	Normal		64	1
ID00849	Normal		64.8	1
ID01482	Normal		64.8	1
ID01483	Normal		64.8	1
ID00229	Normal		64.9	1
ID01484	Normal		65.1	1
ID01485	Normal		65.2	1
ID01486	Normal		65.5	1
ID01487	Normal		65.5	1
ID01488	Normal		66	1
ID01489	Normal		66.2	1
ID01490	Normal		66.7	1
ID01491	Normal		68	1
ID00420	Normal		68.2	1
ID01107	Normal		68.4	1
ID00383	Normal		68.7	1
ID01492	Normal		68.7	1
ID01493	Normal		68.7	1
ID01494	Normal		68.8	1
ID00462	Normal		69	1
ID01495	Normal		69	1
ID01496	Normal		69.1	1
ID01497	Normal		69.5	1
ID01498	Normal		69.6	1
ID00868	Normal		69.7	1
ID01499	Normal		70	1
ID01240	Normal		70.2	1
ID01500	Normal		70.3	1
ID01501	Normal		70.4	1
ID01502	Normal		70.5	1
ID01503	Normal		70.55	1
ID01504	Normal		70.7	1

ID01505		Normal		70.8	1
ID01506		Normal		70.9	1
ID01507		Normal		71.3	1
ID01508		Normal		71.4	1
ID00491		Normal		71.6	1
ID01509		Normal		71.6	1
ID01510		Normal		71.7	1
ID01511		Normal		71.8	1
ID01512		Normal		72.1	1
ID00621		Normal		72.55	1
ID01513		Normal		72.7	1
ID01514		Normal		72.9	1
ID01515		Normal		72.9	1
ID01516		Normal		73.7	1
ID01517		Normal		73.9	1
ID00828		Normal		74.1	1
ID01518		Normal		74.1	1
ID01519		Normal		74.6	1
ID01520		Normal		74.6	1
ID01521		Normal		74.6	1
ID00063	Normal	Normal	41.6	74.7	2
ID01522		Normal		74.75	1
ID01523		Normal		74.8	1
ID01524		Normal		74.8	1
ID00837	Normal	Normal	75.5	75.5	2
ID00601		Normal		75.6	1
ID01525		Normal		75.6	1
ID01526		Normal		76.2	1
ID01527		Normal		76.4	1
ID01528		Normal		76.8	1
ID01529		Normal		77.3	1
ID01530		Normal		77.3	1
ID00438		Normal		77.8	1
ID00339		Normal		78.1	1
ID01531		Normal		78.2	1
ID01271		Normal		78.3	1
ID01532		Normal		78.4	1
ID01533		Normal		78.6	1
ID01534		Normal		78.8	1
ID00426		Normal		78.9	1
ID00785		Normal		79.1	1
ID00891		Normal		79.1	1
ID01535		Normal		79.1	1
ID01536		Normal		79.9	1
ID00233		Normal		80.1	1
ID01537		Normal		80.7	1
ID01538		Normal		80.8	1
ID01539		Normal		81.3	1
ID01540		Normal		81.8	1
ID01541		Normal		82.2	1

ID00786		Normal		82.3	1
ID01542		Normal		82.4	1
ID01543		Normal		82.4	1
ID01544		Normal		82.4	1
ID01545		Normal		82.4	1
ID01015		Normal		82.5	1
ID01546		Normal		82.9	1
ID01547		Normal		82.9	1
ID01548		Normal		83.2	1
ID01549		Normal		83.5	1
ID00336		Normal		83.6	1
ID01133		Normal		84	1
ID00659		Normal		84.2	1
ID01550		Normal		84.3	1
ID01551		Normal		84.4	1
ID00033		Normal		84.6	1
ID01552		Normal		84.6	1
ID01553		Normal		85.1	1
ID01258		Normal		85.4	1
ID01554		Normal		85.5	1
ID01555		Normal		85.6	1
ID01556		Normal		85.6	1
ID01557		Normal		85.7	1
ID01558		Normal		85.9	1
ID01559		Normal		86	1
ID01560		Normal		86.45	1
ID01561		Normal		86.7	1
ID01562		Normal		87	1
ID01563		Normal		87.3	1
ID01098		Normal		87.7	1
ID01564		Normal		88.4	1
ID01565		Normal		88.4	1
ID01566		Normal		88.55	1
ID00823		Normal		88.6	1
ID00876		Normal		88.7	1
ID01286		Normal		88.7	1
ID01567		Normal		88.8	1
ID01568		Normal		89.1	1
ID01569		Normal		90.1	1
ID00941		Normal		90.2	1
ID01570		Normal		90.7	1
ID01571		Normal		90.8	1
ID00877		Normal		91.2	1
ID01572		Normal		91.7	1
ID01573		Normal		91.9	1
ID01574		Normal		92.2	1
ID01087		Normal		92.4	1
ID00530	Normal	Normal	59	92.5	2
ID01575		Normal		92.7	1
ID01576		Normal		92.9	1

ID01577	Normal	93.4	1
ID01578	Normal	93.6	1
ID01579	Normal	93.6	1
ID01580	Normal	93.7	1
ID00595	Normal	93.9	1
ID01581	Normal	94.3	1
ID01582	Normal	94.7	1
ID01583	Normal	95.4	1
ID01584	Normal	95.6	1
ID01585	Normal	95.7	1
ID00940	Normal	96.1	1
ID01586	Normal	96.2	1
ID01587	Normal	96.3	1
ID00600	Normal	96.4	1
ID01588	Normal	96.5	1
ID01589	Normal	96.8	1
ID01590	Normal	96.9	1
ID01591	Normal	97	1
ID00081	Normal	97.05	1
ID00218	Normal	98	1
ID00683	Normal	98.4	1
ID01592	Normal	98.4	1
ID01593	Normal	98.8	1
ID01594	Normal	98.9	1
ID01595	Normal	99	1
ID01596	Normal	99.3	1
ID01597	Normal	99.4	1
ID01598	Normal	99.7	1
ID01599	Normal	99.9	1
ID01255	Normal	100	1
ID01600	Normal	100	1
ID01601	Normal	100	1
ID01602	Normal	100	1
ID01603	Normal	100	1
ID01604	Normal	101	1
ID00211	Normal	101.05	1
ID00089	Normal	102	1
ID00216	Normal	102	1
ID01605	Normal	102	1
ID00250	Normal	103	1
ID01264	Normal	103	1
ID01606	Normal	103	1
ID01607	Normal	103	1
ID01608	Normal	103	1
ID01609	Normal	103	1
ID01610	Normal	104	1
ID01611	Normal	104	1
ID01612	Normal	104	1
ID01613	Normal	104	1
ID01614	Normal	104	1

ID01615	Normal		104	1
ID01616	Normal		104	1
ID01617	Normal		105	1
ID01618	Normal		105	1
ID01619	Normal		105	1
ID01620	Normal		106	1
ID01621	Normal		106	1
ID01622	Normal		106	1
ID01623	Normal		106.8	1
ID01624	Normal		107	1
ID01625	Normal		107	1
ID01626	Normal		107	1
ID00179	Normal		109	1
ID00241	Normal		109	1
ID00077	Normal		110	1
ID00615	Normal		110	1
ID00626	Normal		110	1
ID01627	Normal		110	1
ID01628	Normal		110	1
ID01629	Normal		110	1
ID00258	Normal		111	1
ID00524	Normal		111	1
ID01630	Normal		111	1
ID01631	Normal		111	1
ID01632	Normal		111	1
ID01633	Normal		111	1
ID01634	Normal		111	1
ID01635	Normal		111	1
ID01636	Normal		111	1
ID01637	Normal		112	1
ID01638	Normal		112.5	1
ID00498	Normal		113	1
ID00775	Normal		113	1
ID01153	Normal		113	1
ID01639	Normal		113	1
ID01640	Normal		113	1
ID01641	Normal		113	1
ID01642	Normal		114	1
ID01643	Normal		114	1
ID00604	Normal		115	1
ID01185	Normal		115	1
ID01644	Normal		115	1
ID01645	Normal		117	1
ID01646	Normal		119	1
ID00222	Normal		120	1
ID00688	Normal		120	1
ID01647	Normal		120	1
ID01648	Normal		120	1
ID01649	Normal		120	1
ID01650	Normal		120	1

ID00560		Normal		121	1
ID00618		Normal		121	1
ID00817		Normal		121	1
ID00903		Normal		121	1
ID01651		Normal		121	1
ID01652		Normal		121	1
ID01653		Normal		121	1
ID00107		Normal		122	1
ID01654		Normal		122	1
ID01655		Normal		123	1
ID01656		Normal		123	1
ID01249		Normal		124	1
ID01657		Normal		124	1
ID00657		Normal		125	1
ID01658		Normal		125	1
ID01659		Normal		125	1
ID01001		Normal		127	1
ID01660		Normal		127	1
ID00781	Gray Zone	Normal	28.7	128	2
ID01661		Normal		128	1
ID01662		Normal		129	1
ID01663		Normal		129	1
ID00238		Normal		130	1
ID00928		Normal		130	1
ID01664		Normal		130	1
ID01665		Normal		130	1
ID00872		Normal		131	1
ID00761		Normal		132	1
ID01666		Normal		132	1
ID01667		Normal		133	1
ID01668		Normal		134	1
ID01669		Normal		134	1
ID01670		Normal		137	1
ID01671		Normal		137	1
ID01672		Normal		139	1
ID01673		Normal		139	1
ID01674		Normal		140	1
ID00729	Normal	Normal	62.6	141	2
ID01675		Normal		141	1
ID01676		Normal		143	1
ID01677		Normal		143	1
ID00255		Normal		144	1
ID00749		Normal		144	1
ID01678		Normal		144	1
ID01679		Normal		144	1
ID00481		Normal		145	1
ID01101		Normal		147	1
ID01680		Normal		147	1
ID01681		Normal		147.5	1
ID01682		Normal		149	1

ID01683		Normal		149.5	1
ID01270		Normal		150	1
ID01684		Normal		151	1
ID01685		Normal		151	1
ID00911		Normal		152.5	1
ID00611		Normal		154	1
ID01686		Normal		154	1
ID01687		Normal		156	1
ID01055		Normal		159	1
ID01688		Normal		159	1
ID01689		Normal		159	1
ID01078		Normal		160	1
ID01690		Normal		160	1
ID00257		Normal		163	1
ID01691		Normal		163	1
ID01692		Normal		163	1
ID01693		Normal		164	1
ID01242		Normal		165	1
ID01694		Normal		165	1
ID01263		Normal		166	1
ID01695		Normal		167	1
ID01696		Normal		170	1
ID00061		Normal		172	1
ID01697		Normal		178	1
ID01698		Above normal range		182	1
ID01002		Above normal range		191	1
ID01699		Above normal range		191.5	1
ID01700		Above normal range		196	1
ID01701		Above normal range		205	1
ID01702		Above normal range		212.6	1
ID01703		Above normal range		220.7	1
ID01704		Above normal range		234	1
ID01705		Above normal range		234	1
ID01706		Above normal range		247	1
ID01707		Above normal range		253	1
ID01708		Above normal range		253	1
ID01709		Above normal range		283	1
ID01188	Adult		0.378		1
ID00693	Adult		0.499		1
ID01303	Unknown/Deficient		0.885		1
ID01096	Adult		0.991		1
ID01142	Adult		1.05		1
ID00541	Adult		1.31		1
ID01029	Childhood		1.5		1
ID00512	Adult		1.71		1
ID01189	Adult		2.06		1
ID00025	Adult		2.25		1
ID01046	Adult		2.54		1
ID00150	Childhood		2.6		1
ID00547	Adult		3		1

ID00422	Adult	3.31	1
ID01304	Unknown/Deficient	3.67	1
ID00292	Adult	4.3	1
ID00632	Adult	4.73	1
ID00153	Adult	5	1
ID00906	Adult	8.2	1
ID01305	Gray Zone	16.3	1
ID00104	Gray Zone	22.3	1
ID00180	Gray Zone	25.85	1
ID00559	Gray Zone	30.9	1
ID00557	Gray Zone	31.5	1
ID00005	Gray Zone	34.2	1
ID00201	Gray Zone	36	1
ID01155	Gray Zone	36.8	1
ID01306	Gray Zone	37.6	1
ID00435	Gray Zone	38	1
ID00558	Gray Zone	38.3	1
ID00160	Gray Zone	38.8	1
ID01307	Gray Zone	39.3	1
ID00011	Gray Zone	39.8	1
ID00231	Normal	40.5	1
ID00959	Normal	40.8	1
ID01215	Normal	41.2	1
ID00556	Normal	42.9	1
ID00299	Normal	44.6	1
ID00942	Normal	45.1	1
ID00269	Normal	45.9	1
ID01170	Normal	46.4	1
ID00190	Normal	46.6	1
ID01067	Normal	46.6	1
ID01194	Normal	47	1
ID00430	Normal	47.3	1
ID00755	Normal	47.3	1
ID00315	Normal	48.2	1
ID01199	Normal	48.7	1
ID01163	Normal	49	1
ID00094	Normal	49.1	1
ID00802	Normal	49.3	1
ID00625	Normal	50.1	1
ID00432	Normal	51	1
ID01308	Normal	51.35	1
ID00784	Normal	51.9	1
ID01044	Normal	53.3	1
ID00285	Normal	53.4	1
ID00037	Normal	54.1	1
ID01052	Normal	54.6	1
ID00695	Normal	54.7	1
ID01309	Normal	55.1	1
ID00831	Normal	56.3	1
ID00433	Normal	56.9	1

ID01310	Normal	58.5	1
ID00484	Normal	58.9	1
ID00832	Normal	59.2	1
ID00900	Normal	59.4	1
ID00662	Normal	59.5	1
ID00447	Normal	60.4	1
ID00691	Normal	60.5	1
ID00417	Normal	60.9	1
ID00922	Normal	61.4	1
ID00569	Normal	61.6	1
ID00295	Normal	61.9	1
ID00283	Normal	62.2	1
ID00001	Normal	62.4	1
ID00404	Normal	63.4	1
ID00169	Normal	63.5	1
ID00181	Normal	63.5	1
ID00418	Normal	63.6	1
ID00095	Normal	64.1	1
ID00397	Normal	64.3	1
ID01273	Normal	64.65	1
ID01311	Normal	64.7	1
ID01175	Normal	64.8	1
ID00284	Normal	65	1
ID00516	Normal	65.3	1
ID00184	Normal	65.4	1
ID01045	Normal	65.8	1
ID00296	Normal	66.7	1
ID01287	Normal	66.7	1
ID00364	Normal	67	1
ID00937	Normal	67.1	1
ID00406	Normal	68.5	1
ID01186	Normal	69	1
ID01202	Normal	70.1	1
ID00403	Normal	70.2	1
ID00282	Normal	70.6	1
ID00267	Normal	71.4	1
ID00705	Normal	71.9	1
ID00833	Normal	72.3	1
ID01119	Normal	72.9	1
ID00313	Normal	73	1
ID00529	Normal	73.9	1
ID01106	Normal	74.3	1
ID00117	Normal	74.4	1
ID00917	Normal	74.4	1
ID00410	Normal	75.2	1
ID00915	Normal	75.2	1
ID00504	Normal	75.3	1
ID01104	Normal	75.3	1
ID00128	Normal	75.5	1
ID00591	Normal	75.6	1

ID00157	Normal	75.8		1
ID00531	Normal	76.7		1
ID00800	Normal	77.2		1
ID00562	Normal	77.5		1
ID01024	Normal	77.8		1
ID00209	Normal	78.5		1
ID00306	Normal	78.9		1
ID00946	Normal	79		1
ID00307	Normal	79.4		1
ID00402	Normal	79.6		1
ID00676	Normal	79.9		1
ID00692	Normal	79.9		1
ID01139	Normal	80.1		1
ID00935	Normal	80.2		1
ID00278	Normal	80.6		1
ID00020	Normal	80.7		1
ID00656	Normal	80.9		1
ID00702	Normal	81		1
ID00429	Normal	81.1		1
ID00023	Normal	81.8		1
ID00919	Normal	81.8		1
ID01177	Normal	82.2		1
ID00521	Normal	82.7		1
ID00720	Normal	83.3		1
ID00620	Normal	83.4		1
ID00187	Normal	83.7		1
ID00413	Normal	84.1		1
ID00144	Normal	84.2		1
ID00210	Normal	84.2		1
ID00948	Normal	85.2		1
ID01312	Normal	85.6		1
ID00811	Normal	85.8		1
ID01313	Normal	85.8		1
ID00851	Normal	86.7		1
ID00006	Normal	87.1		1
ID00949	Normal	87.6		1
ID00555	Normal	88.1		1
ID00574	Normal	88.2		1
ID00543	Normal	88.3		1
ID00986	Normal	89.1		1
ID00112	Normal	89.6		1
ID00118	Normal	89.8		1
ID00388	Normal	89.9		1
ID01076	Normal	89.9		1
ID00794	Normal	90		1
ID01014	Normal	90.6		1
ID00544	Normal	92.1		1
ID00805	Normal	92.5		1
ID01180	Normal	92.6		1
ID01203	Normal	92.7		1

ID00550	Normal	92.8		1
ID00856	Normal	93.2		1
ID00390	Normal	93.3		1
ID00016	Normal	93.7		1
ID01314	Normal	93.7		1
ID00535	Normal	93.9		1
ID00386	Normal	94		1
ID00102	Normal	94.2		1
ID01204	Normal	95.3		1
ID00797	Normal	95.7		1
ID01289	Normal	96.1		1
ID00645	Normal	96.9		1
ID01178	Normal	97.1		1
ID01315	Normal	97.6		1
ID00186	Normal	97.7		1
ID00105	Normal	98.6		1
ID01027	Normal	98.7		1
ID00162	Normal	98.8		1
ID01035	Normal	99		1
ID00448	Normal	99.4		1
ID00796	Normal	99.8		1
ID00093	Normal	100		1
ID00365	Normal	100		1
ID00549	Normal	100		1
ID00819	Normal	100		1
ID00479	Normal	101		1
ID00513	Normal	101		1
ID00816	Normal	101		1
ID00818	Normal	102		1
ID00943	Normal	102		1
ID00027	Normal	103		1
ID00223	Normal	103		1
ID00270	Normal	103		1
ID00532	Normal	103		1
ID00858	Normal	103		1
ID01042	Normal	103		1
ID01148	Normal	103		1
ID00916	Normal	104.4		1
ID00703	Normal	104.5		1
ID00166	Normal	105		1
ID00952	Normal	105		1
ID00436	Normal	106		1
ID00446	Normal	107		1
ID00590	Normal	107		1
ID00826	Normal	107		1
ID00177	Normal	108		1
ID00188	Normal	108		1
ID00431	Normal	108		1
ID00419	Normal	109		1
ID00538	Normal	109		1

ID01316	Normal	109	1
ID00260	Normal	110	1
ID00696	Normal	110	1
ID00933	Normal	110	1
ID01156	Normal	110	1
ID01288	Normal	110	1
ID00212	Normal	111	1
ID00303	Normal	111	1
ID00814	Normal	111	1
ID00100	Normal	112	1
ID00566	Normal	112	1
ID01205	Normal	112	1
ID00106	Normal	113	1
ID01122	Normal	113	1
ID01154	Normal	113	1
ID00405	Normal	114	1
ID00664	Normal	114	1
ID00821	Normal	114	1
ID00788	Normal	115	1
ID00553	Normal	116	1
ID01003	Normal	116	1
ID01232	Normal	116	1
ID00536	Normal	117	1
ID00863	Normal	117	1
ID01060	Normal	117	1
ID00293	Normal	118	1
ID00300	Normal	118	1
ID01025	Normal	118	1
ID01061	Normal	118	1
ID01317	Normal	118	1
ID00686	Normal	119	1
ID00827	Normal	119	1
ID00883	Normal	119	1
ID00993	Normal	119	1
ID01190	Normal	119	1
ID01056	Normal	120	1
ID00502	Normal	121	1
ID01021	Normal	121	1
ID00309	Normal	123	1
ID01048	Normal	123	1
ID00421	Normal	124	1
ID00423	Normal	124	1
ID00809	Normal	124	1
ID00191	Normal	125	1
ID00434	Normal	125	1
ID01108	Normal	125	1
ID00014	Normal	126	1
ID00035	Normal	126	1
ID00116	Normal	126	1
ID01318	Normal	126	1

ID00002	Normal	127		1
ID00168	Normal	127		1
ID00287	Normal	127		1
ID00932	Normal	127		1
ID00445	Normal	128		1
ID01064	Normal	129		1
ID01219	Normal	129		1
ID00234	Normal	130		1
ID00545	Normal	130		1
ID00921	Normal	130		1
ID00031	Normal	131		1
ID01008	Normal	131		1
ID01200	Normal	131		1
ID01319	Normal	131		1
ID00024	Normal	132		1
ID00673	Normal	132		1
ID00799	Normal	132		1
ID01238	Normal	132		1
ID01320	Normal	132		1
ID00759	Normal	133		1
ID01290	Normal	133		1
ID00741	Normal	136		1
ID01043	Normal	136		1
ID01256	Normal	136		1
ID00638	Normal	136.5		1
ID00400	Normal	137		1
ID00650	Normal	137		1
ID00007	Normal	138		1
ID00414	Normal	139		1
ID01050	Normal	139		1
ID01179	Normal	139		1
ID01321	Normal	139		1
ID00647	Normal	140		1
ID00401	Normal	143		1
ID00694	Normal	143		1
ID01322	Normal	143		1
ID00026	Normal	144		1
ID00316	Normal	144		1
ID00428	Normal	145		1
ID01323	Normal	145		1
ID00178	Normal	146		1
ID00394	Normal	146		1
ID00605	Normal	146		1
ID00511	Normal	147		1
ID00631	Normal	147		1
ID00944	Normal	147		1
ID00463	Normal	149		1
ID01109	Normal	149		1
ID00561	Normal	150		1
ID00866	Normal	150		1

ID00920	Normal	150	1
ID00167	Normal	151	1
ID00344	Normal	151	1
ID00302	Normal	152	1
ID01147	Normal	152	1
ID00399	Normal	153	1
ID00182	Normal	154	1
ID00893	Normal	157	1
ID01168	Normal	157	1
ID00587	Normal	159	1
ID00003	Normal	160	1
ID00119	Normal	162	1
ID01197	Normal	162	1
ID00173	Normal	165	1
ID00918	Normal	165	1
ID00539	Normal	167	1
ID00912	Normal	168	1
ID00934	Normal	168	1
ID00407	Normal	169	1
ID00552	Normal	172	1
ID00573	Normal	172	1
ID00654	Normal	172	1
ID01132	Normal	172	1
ID00228	Normal	173	1
ID01059	Normal	173	1
ID00563	Normal	174	1
ID00057	Normal	176	1
ID00192	Normal	176	1
ID00194	Normal	176	1
ID01192	Normal	176	1
ID00099	Normal	177	1
ID00425	Normal	177	1
ID01195	Normal	185	1
ID00022	Normal	187	1
ID00929	Normal	187	1
ID00444	Normal	188	1
ID00318	Normal	195	1
ID00437	Normal	196	1
ID01191	Normal	197	1
ID01066	Normal	200	1
ID01058	Normal	203	1
ID00236	Normal	211	1
ID00824	Normal	212	1
ID00305	Normal	214	1
ID01138	Normal	214	1
ID00679	Normal	216	1
ID00822	Normal	217	1
ID00185	Normal	217.5	1
ID00698	Normal	220	1
ID00277	Normal	221	1

ID00808	Normal	223		1
ID00671	Normal	225		1
ID00159	Normal	231		1
ID01324	Normal	239		1
ID01325	Normal	246		1
ID00674	Normal	248		1
ID01054	Above normal range	256		1
ID00666	Above normal range	265		1
ID00009	Above normal range	271		1
ID00938	Above normal range	279		1
ID00947	Above normal range	299		1
ID01326	Above normal range	341		1
				2591
